# Msa1 and Msa2 Modulate G1-Specific Transcription to Promote G1 Arrest and the Transition to Quiescence in Budding Yeast

**DOI:** 10.1371/journal.pgen.1006088

**Published:** 2016-06-06

**Authors:** Shawna Miles, Matthew W. Croxford, Amali P. Abeysinghe, Linda L. Breeden

**Affiliations:** Basic Science Division, Fred Hutchinson Cancer Research Center, Seattle, Washington, United States of America; University of Massachusetts Medical School, UNITED STATES

## Abstract

Yeast that naturally exhaust their glucose source can enter a quiescent state that is characterized by reduced cell size, and high cell density, stress tolerance and longevity. The transition to quiescence involves highly asymmetric cell divisions, dramatic reprogramming of transcription and global changes in chromatin structure and chromosome topology. Cells enter quiescence from G1 and we find that there is a positive correlation between the length of G1 and the yield of quiescent cells. The Swi4 and Swi6 transcription factors, which form the SBF transcription complex and promote the G1 to S transition in cycling cells, are also critical for the transition to quiescence. Swi6 forms a second complex with Mbp1 (MBF), which is not required for quiescence. These are the functional analogues of the E2F complexes of higher eukaryotes. Loss of the RB analogue, Whi5, and the related protein Srl3/Whi7, delays G1 arrest, but it also delays recovery from quiescence. Two MBF- and SBF-Associated proteins have been identified that have little effect on SBF or MBF activity in cycling cells. We show that these two related proteins, Msa1 and Msa2, are specifically required for the transition to quiescence. Like the E2F complexes that are quiescence-specific, Msa1 and Msa2 are required to repress the transcription of many SBF target genes, including *SWI4*, the *CLN2* cyclin and histones, specifically after glucose is exhausted from the media. They also activate transcription of many MBF target genes. *msa1msa2* cells fail to G1 arrest and rapidly lose viability upon glucose exhaustion. *msa1msa2* mutants that survive this transition are very large, but they attain the same thermo-tolerance and longevity of wild type quiescent cells. This indicates that Msa1 and Msa2 are required for successful transition to quiescence, but not for the maintenance of that state.

## Author Summary

In spite of the many differences between yeast and humans, the basic strategies that regulate the cell division cycle are fundamentally conserved. In this study, we extend these parallels to include a common strategy by which cells transition from proliferation to quiescence. The decision to divide is made in the G1 phase of the cell cycle. During G1, the genes that drive DNA replication are repressed by the E2F/RB complex. When a signal to divide is received, RB is removed and the complex is activated. When cells commit to a long term, but reversible G1 arrest, or quiescence, they express a novel E2F/RB-like complex, which promotes and maintains a stable repressive state. Yeast cells contain a functional analog of E2F/RB, called SBF/Whi5, which is activated by a similar mechanism in proliferating yeast cells. In this study, we identify two novel components of the SBF/Whi5 complex whose activity is specific to the transition to quiescence. These factors, Msa1 and Msa2, repress SBF targets and are required for the long term, but reversible G1 arrest that is critical for achieving a quiescent state.

## Introduction

The need to stop proliferation and remain in a protected quiescent state is universally conserved and is just as important to yeast as it is to human cells. Failure to enter, or unscheduled exit from quiescence results in uncontrolled proliferation and cancer in humans, and death in unicellular organisms [1]. Most cells enter quiescence from G1. As such, there must be regulators in G1 cells capable of recognizing stop signals when they arise and provoking a stable but reversible halt to S phase. The regulatory strategy that controls the G1 to S transition in cycling cells is well understood and its basic framework is highly conserved from yeast to humans [2]. Studies of yeast have provided many insights into this process, but little is known about the cell cycle regulators that give rise to quiescent yeast cells. We have identified a pair of related transcription factors that play a critical role in halting the cell cycle in G1, specifically during the transition to quiescence. Like the highly conserved quiescence-specific complexes of higher eukaryotes [3–5], these factors repress transcripts that promote the G1 to S transition and enable yeast cells to enter the quiescent state.

In rapidly growing yeast cells, as in higher cells, the G1 to S transition is tightly controlled by two consecutive waves of cyclin expression. Cln3 is expressed at the M/G1 boundary and initiates the transition by binding and activating the cyclin-dependent kinase (Cdk). The critical target of Cln3/Cdk is Whi5, which represses SBF. SBF is a transcription factor complex that includes Swi6 and its DNA binding partner Swi4. Cln3 phosphorylates and releases Whi5 from the complex, thus enabling SBF to activate late G1-specific transcription of the G1 cyclins *CLN1* and *CLN2* and other genes that promote the G1 to S transition [6–8]. The G1 cyclin/Cdk complexes then phosphorylate Sic1 and target it for degradation. Once Sic1 is degraded, the B type cyclin/Cdk complexes that are bound and inhibited by Sic1 are released, allowing them to phosphorylate and activate the DNA replication machinery and S phase ensues.

Swi6 also associates with a second DNA binding protein, Mbp1, which is related to Swi4 and binds to a similar but distinct DNA sequence [9]. This complex, referred to as MBF, also confers late-G1 specific transcription on many genes involved in DNA replication and repair. These genes are regulated by Nrm1-dependent negative feedback [10]. Nrm1, itself a late-G1 transcript, accumulates in S phase, binds MBF complexes and represses transcription through S and G2/M.

This wave of late G1 transcription is critical for the timing and fidelity of DNA replication. If the G1 to S transition is accelerated by ectopic expression of Swi4 or the G1 cyclin Cln2, there are checkpoint proteins, including Mec1 and Rad53, that detect replication stress and become essential for delaying S phase and promoting DNA repair [11, 12]. This is in part accomplished by the direct phosphorylation of Nrm1 by Rad53, which releases it from the MBF complex and allows DNA replication and repair genes to be activated [13].

The transition from logarithmic growth to quiescence involves a stable but reversible cell cycle arrest in G1. Our previous studies have shown that this transition begins with a lengthening of G1, which is initiated before the diauxic shift (DS), when all the glucose has been taken up from the media [14]. The cell divisions that follow are highly asymmetric and the physical growth of those cells slows, resulting in a dramatic shift in the cell size of the population [15]. To explore the mechanism of this stable but reversible G1 arrest associated with quiescence, we have assessed the roles of known regulators of the G1 to S transition in rapidly growing cells. In wild type cells, Rad53 plays a role in the transition to quiescence, and it becomes essential if the G1 to S transition is driven by Cln3 overproduction. A second checkpoint gene, Rad9, is not required during this transition. Rad9 responds to DNA damage, while Rad53 responds to both DNA damage and replicative stress. This indicates that the latter, replicative stress, is the likely signal for Rad53 activation during the transition to quiescence [14]. The G1 arrest is maintained in post-diauxic cells by Xbp1, which is induced to high levels and represses *CLN3* along with over 800 other genes [14]. Xbp1 recruits the histone deacetylase, Rpd3, which plays a unique and prominent role in the transcriptional repression that takes place in quiescent cells [16]. Rpd3 is targeted to at least half the budding yeast promoters, where it affects global nucleosome repositioning, histone deacetylation and a 30-fold global repression of transcription [16].

In this paper, we report the role of two proteins, Msa1 and Msa2 in the early transcriptional regulation that promotes G1 arrest and the transition to quiescence. Msa1 and Msa2 are two related proteins that were identified by mass spectrometry to be associated with SBF and MBF complexes [17]. Mutations in these proteins have mild phenotypes in rapidly growing cells [17–19], but we find that Msa1 and Msa2 are both important during the transition to quiescence. Each single mutant survives this transition, but the *msa1msa2* double mutant fails to G1 arrest and loses viability rapidly. When paired with *rad53-21*, we observe a G1 arrest defect with the single mutants, especially *msa2*. These data indicate that Msa1 and Msa2 are both important regulators that promote G1 arrest during the transition to quiescence and cells rely on the Rad53 checkpoint function when either protein is missing. We have carried out RNA deep sequencing with the single and double *msa* mutants as cells transition from log phase to quiescence and find that they have significant impact on the expression of both MBF and SBF target genes, specifically in post-diauxic cells. In many cases, both Msa1 and Msa2 are required to repress SBF targets and activate MBF targets, and their effects are not additive. This suggests that they both play critical roles in the regulation of these late G1-specific transcripts. In other contexts, either Msa protein is sufficient to perform their regulatory function in post-DS cells. Chromatin immunoprecipitation (ChIP) of candidate targets show binding of both Msa1 and Msa2 to their targets, and show stronger binding in post-DS cells. The post-diauxic regulation of these genes by Msa1 and Msa2 is likely to be important for a normal transition from proliferation to quiescence.

## Results

Quiescent cells can be purified from stationary phase cultures due to their high density [20]. These quiescent (Q) cells, by definition, are in a stable, long-lived, but reversible arrest, and they have G1 DNA content, suggesting that they exit the cell cycle from G1. It follows that regulators that promote the G1 to S transition, might disrupt entry into quiescence and those that prolong G1 might facilitate it. This is clearly true of the activator Cln3, which in excess reduces Q cell yield and when absent increases Q cell yield [14] and Table 1).

**Table 1:** Relationship between the length of G1 in log phase and high density Q cell yield.

Strain		%G1	SD	%Q	SD
BY5654	*5xCLN3*	6.8	1.5	26	5.4
BY6828	*sic1*	6.8	0.0	19	2.3
BY6698	*5xCLN3rad53-21*	7.2	0.9	1.4	0.0
BY7002	*swi6*	7.8	4.3	0.1	0.2
BY7326	*whi5*	10.3	0.5	45.2	2.4
BY7794	*stb1whi5*	11.5	0.2	40.0	3.6
BY7334	*whi5srl3*	12.3	0.4	46.6	0.8
BY7140	*msa2rad53-21*	13.0	0.3	54.7	2.1
BY7171	*msa2rad53-21*				
BY6848	*rad53-21*	14.5	5.0	52	4.4
BY7793	*stb1msa2*	17.5	2.1	40.8	8.0
BY7067	*msa1rad53-21*	17.9	3.2	62	1.3
BY7448	*whi5msa1*	17.9	0.2	56.7	4.9
BY7795	*stb1msa1*	17.9	0.2	38.9	0.4
BY7146	*xbp1rad53-21*	18.6	1.0	46	2.6
BY6884	*stb1*	19.1	0.5	62	1.6
BY6879	*msa1*	19.2	0.7	61	2.9
BY6966	*hog1*	19.2	0.2	51	8.1
BY6883	*msa1msa2*	19.6	1.6	34	6.4
BY6602	*xbp1*	20.1	0.9	54	3.7
BY7332	*srl3*	21.0	1.0	62.1	1.9
BY7454	*whi5msa2*	21.1	0.5	68.6	2.0
BY6500	WT	21.2	2.1	56	8.6
BY6881	*msa2*	21.3	0.9	57	1.5
BY7131	*xbp1cln3*	28.4	0.0	60	1.5
BY7026	*mbp1*	29.0	0.7	53	2.5
BY6873	*cln3*	34.3	1.9	70	1.2

Strains are all isogenic with prototrophic W303 (BY6500) with relevant genotype listed. Percent of cells in G1 during log phase and the percent yield of high density (Q) cells after seven days of growth are listed, with standard deviations (SD) calculated from 2 to 6 measurements, except *sic1* and *xbp1cln3*, which were only measured once. Two independently derived *msa2rad53-21* strains were analyzed because of the variability observed with this strain. Swi4 is not included because it is lethal in the BY6500 background.

**Fig 1:**
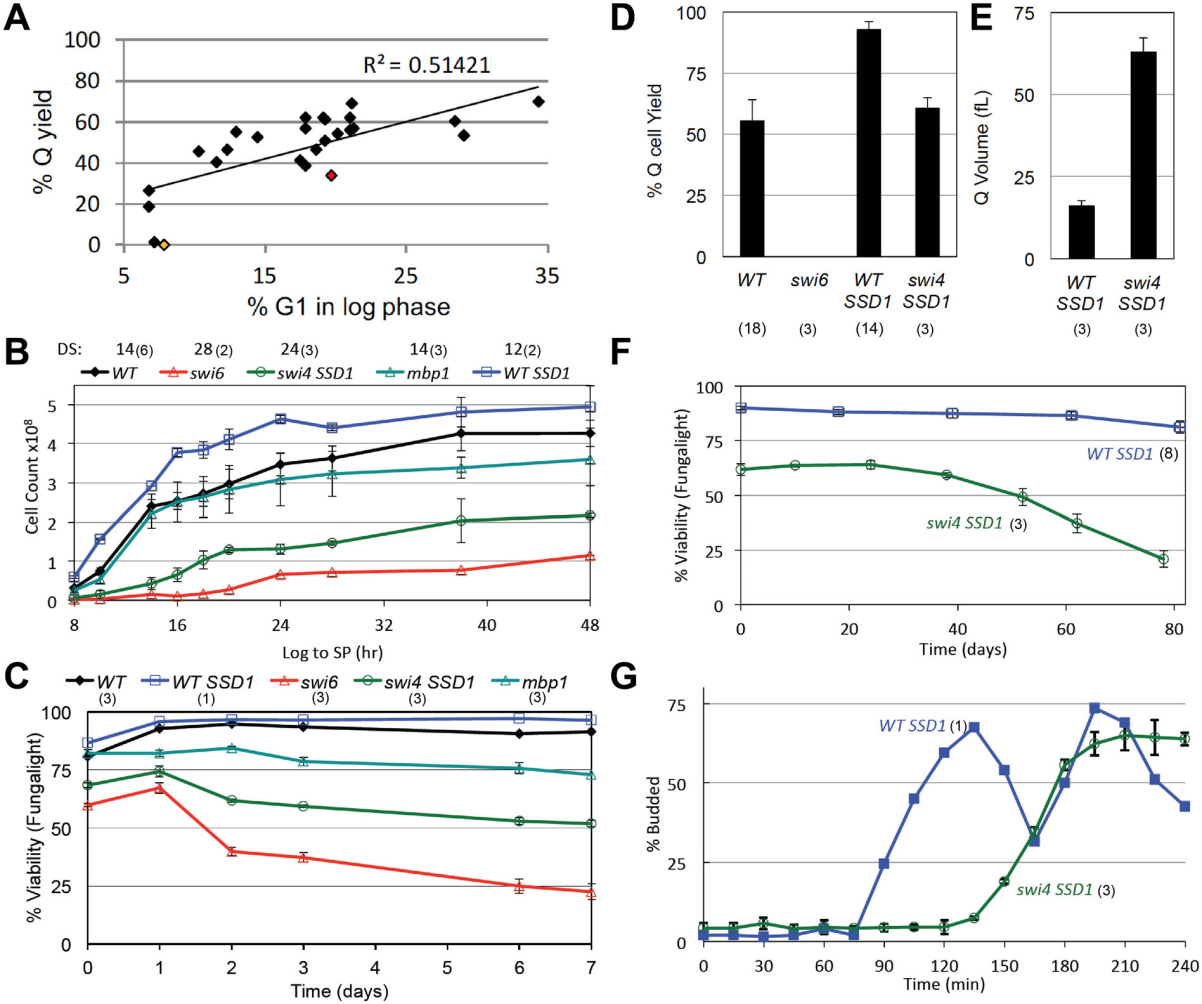
Quiescent cell yield is proportional to the length of time cells spend in G1 during logarithmic growth. (A) Percent yield of high density (Q) cells from cultures grown in YEPD medium for seven days is plotted against the fraction of the population that is in G1 during the log phase of growth. These data and the mutants assayed are listed in Table 1. The *msa1msa2* and *swi6* points are highlighted in red and yellow, respectively. (B) Swi4 and Swi6 activities are critical for cell proliferation as cells grow in YEPD medium from log phase (Log) to stationary phase (SP.) Cell count over 48 hours of growth was determined with a Coulter Counter as described in methods. The hour after which the diauxic shift (DS) has occurred is shown above each genotype. (C) Viability as cells grow over a seven day time course in rich glucose media is assayed by dye exclusion (FungaLight.) All strains are isogenic with prototrophic W303 (BY6500) except *swi4*, which is lethal in this background. In that case, WT *SSD1* (BY6641), which is otherwise isogenic with W303 serves as wild type control (WT) for the *SSD1 swi4*::*KanMx* strain (BY7122). (D) Yield of high density (Q) cells and (E) Q cell volume for the genotypes indicated. (F) Purified *swi4* and WT Q cells from a seven day culture were incubated in water for 80 days and monitored for loss of viability as described in methods. (G) Purified *swi4* and WT Q cells were released into fresh medium and their cell cycle re-entry was monitored by the percent of budded cells. Number of trials averaged for these figures is shown in parentheses.

We have carried out a survey of mutants that affect the length of G1 and find that there is a good correlation between the length of G1 during logarithmic growth and quiescent cell yield (Fig 1A and Table 1). The longer cells spend in G1, the more efficient is their transition into the quiescent state. This is consistent with the view that cells normally enter quiescence from the G1 phase of the cell cycle. This survey also shows that most of the known regulators of the G1 to S transition during rapid growth are not required for Q cell formation.

### SBF is required for the transition to quiescence

The SBF transcription factor drives the transcription of many genes in late G1, which play important activatory roles in the G1 to S transition. Hence, it would be a likely target of negative regulation as cells enter the stable G1 arrest associated with quiescence. If so, loss of Swi4 or Swi6 activity might promote the transition to quiescence as we’ve seen with loss of Cln3 (Table 1). However, we find that both *swi4* and *swi6* mutants suffer significant loss of viability as they are grown from logarithmic (log) phase to stationary phase (SP). Though not considered an essential gene, *swi6* mutants grow very slowly (Fig 1B). Only 60% of the cells are viable during the logarithmic phase of growth and that drops to 20% after seven days (Fig 1C). *SWI4* is an essential gene in the W303 strain, but that lethality is suppressed by *SSD1* [21]. *SSD1swi4* cells show a similar slow growth and loss of viability pattern. Both *swi6* and *swi4* mutants undergo the diauxic shift (DS) late, at about half the cell number that their respective wild type cells undergo the DS (Fig 1B). However, the optical densities of these mutant cultures are comparable to wild type at the DS (OD_550_ 5 to 6), indicating that cell mass is the key variable for the timing of this transition. Both mutants are larger and more heterogeneous than wild type cells based on light scattering (S1 Fig). Dead cells predominate, based on dye exclusion (Fig 1C) and the accumulation of cell debris on the left margin of their flow cytometry profiles (S1 Fig). These data indicate that normal growth control in response to nutrient limitation requires the activities of both of these key regulators of G1.

Wild type cells that have entered quiescence can be purified based on their density in percoll gradients [20]. No such high density cells can be purified from *swi6* cultures (yellow dot in Fig 1A and 1D). This indicates that Swi6 is critical for the transition to quiescence. In contrast, about half the *swi4 SSD1* cells become dense, but these cells are three times the size of wild type Q cells (Fig 1E) and they include both live and dead cells (Fig 1F). The live high density *swi4* cells suffer a further three-fold loss of viability over the course of an 80 day incubation in water compared to wild type cells which drop very little (Fig 1F). The high density *swi4* cells also recover very slowly upon re-feeding (Fig 1G). We conclude that the longevity of the dense *swi4* cells is compromised. By all these criteria, *swi6* and *swi4* mutants are defective in both the log phase of growth and the transition into and out of quiescence.

### Whi5 and the related protein Srl3 promote cell cycle re-entry from quiescence

**Fig 2:**
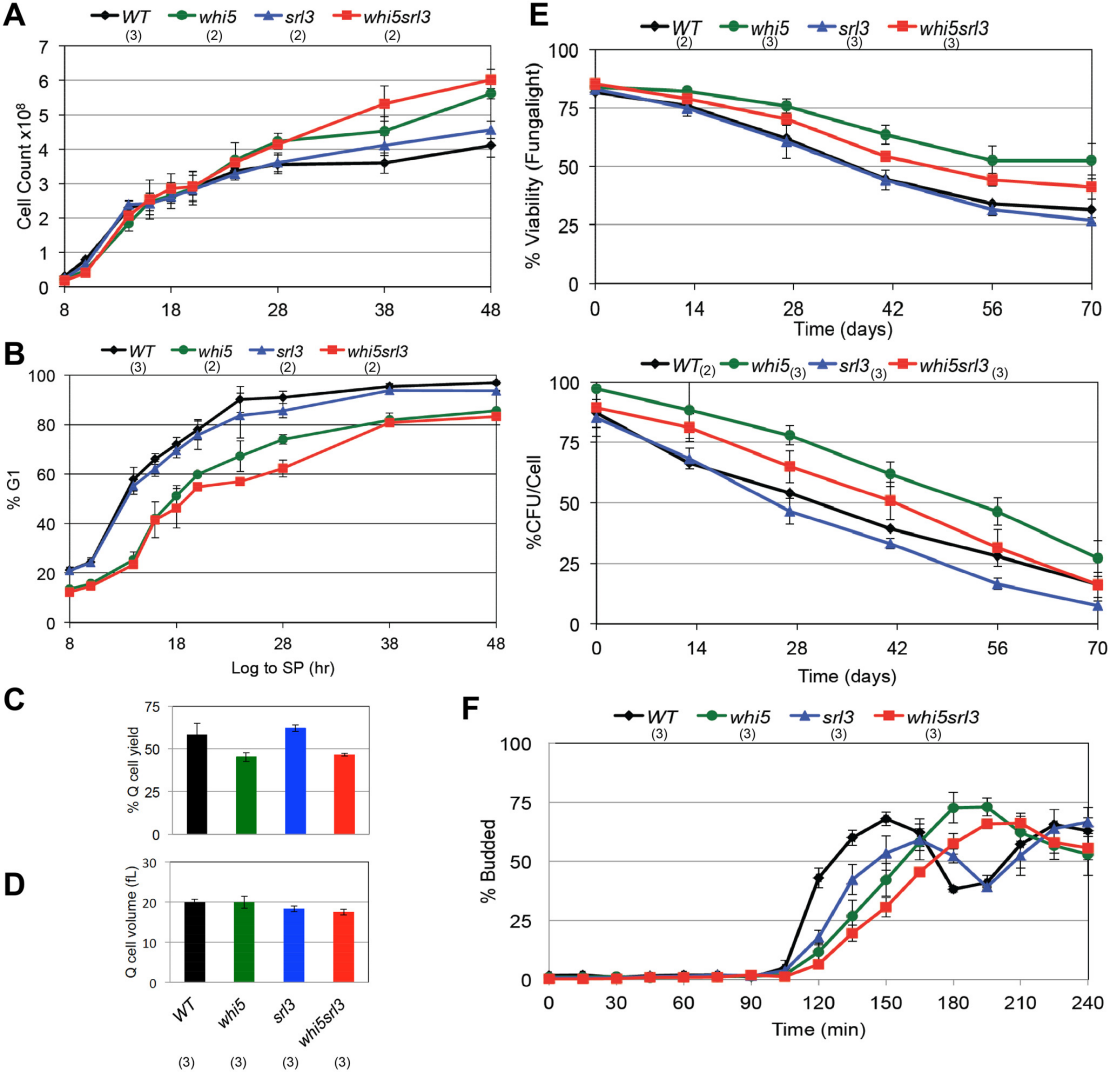
Whi5 and Srl3 are important for the transition out of quiescence. (A) Cell count for the *whi5* and *srl3* mutants as they grow from log phase to stationary phase. (B) Cells over this time course were assayed for DNA content by flow cytometry. Percent of cells with G1 DNA content is plotted. (C) Yield and (D) cell volume of Q cells purified by sedimentation after seven days of growth in rich medium. (E) Longevity of purified Q cells incubated in water at 30°C with aeration was measured by cell viability and percent of colony forming units (CFU). (F) Budding kinetics of Q cells as they re-enter the cell cycle upon re-feeding. Relevant genotypes indicated and strain numbers listed in Table 1. Number of trials averaged for these figures is shown in parentheses.

We have also assayed mutants of other known components of the G1 transcription complexes. The DNA binding component of MBF, Mbp1, is not required for G1 arrest (S1 Fig), viability (Fig 1B and 1C) or for Q cell production (Table 1). Stb1, a component of both SBF and MBF [22–25], is also not required for Q cell production (Table 1). Cells lacking Whi5, which binds and inhibits SBF [6, 7, 25], undergo more cell divisions than wild type (Fig 2A) and they significantly delay, but finally achieve 80% G1 arrest after 48 hours of growth (Fig 2B). The *whi5* mutant produces almost wild type levels of Q cells (Fig 2C), and these cells are identical in size to wild type (Fig 2D). The *whi5* Q cells also have a comparable, if not somewhat longer life span (Fig 2E). This indicates that this SBF repressor plays a role in achieving efficient G1 arrest, but it has no detectable role in the maintenance of quiescent cells. However *whi5* Q cells show a 30 minute delay in recovery from the quiescent state (Fig 2F). This is the opposite of what is seen with G1 cells purified by elutriation, where *whi5* accelerates the transition to S phase and produces smaller cells [6, 7]. These observations suggest that the late G1-specific SBF transcription complex of Swi4 and Swi6 plays a critical role in the transition to quiescence, but that its regulation as cells enter and exit quiescence may involve novel partners other than Whi5.

The whole genome duplication that *S*. *cerevisiae* underwent [26] gave rise to a Whi5-related protein, which was originally identified as a high copy suppressor of *rad53* lethality (*SRL3* [27].) More recently, Srl3 was shown to bind to SBF in response to DNA damage [28] and to regulate the nuclear localization of Cln3 [29]. *SRL3* transcription is induced by DNA damage and many other forms of stress [30–32]. This led us to determine whether Srl3 (also known as Whi7) plays a redundant role with Whi5 in the transition to quiescence. Fig 2 shows that loss of Srl3 causes a modest further delay of G1 arrest, but only when Whi5 is also missing. Its most striking phenotype is the delay of budding as *srl3* and *srl3whi5* Q cells re-enter the cell cycle upon re-feeding.

### Msa1 and Msa2 are critical for G1 arrest and entry into a protective quiescent state

These observations led us to consider two other known components of SBF and MBF transcription complexes. Msa1 and Msa2 are also related proteins that arose from the whole genome duplication. They were initially found by tandem affinity purification and multidimensional protein identification technology (MudPIT) to be associated with both SBF and MBF [17]. Msa1 was also identified as a high copy suppressor of three temperature-sensitive DNA replication mutants [18]. Genome-wide transcript analyses of rapidly growing cells indicated that Msa1 has both an activating and a repressing role at a small and diverse set of target genes during the log phase of growth [18]. The fifty genes identified in that study that are both bound and regulated by Msa1 are mostly involved in glucose metabolism, cell wall organization or ribosomal structure. *MSA1* is an ECB-driven transcript that peaks at the M/G1 boundary, like *CLN3* [33, 34]. Msa1 binds to both SBF and MBF-regulated promoters and has a modest impact on the timing of late G1 transcription and budding in log phase cells [17]. This suggests that Msa1 performs an activatory function at these promoters during the log phase of growth. However, excess Msa1 leads cells to accumulate in G1 and S phase [35], suggesting that Msa1 either represses cell cycle progression directly, or that its presence in excess is activating the DNA damage or replication stress checkpoint. Msa1 also binds to Dbf4, which is the regulatory subunit of the Cdc7/Dbf4 kinase required for DNA replication and for activation of the replication stress checkpoint [36]. More recently, it was shown that the Hog1 kinase phosphorylates Msa1 during osmotic stress, and may play a role in delaying S phase under these conditions [19]. We have not observed a Hog1-dependent effect on the production of quiescent cells (Table 1).

The Msa2 protein sequence is highly conserved compared to that of Msa1. Despite the tight cross-species conservation of Msa2, almost nothing is known about its role in cells. Like, Msa1, it associates with SBF and MBF [17]. *MSA2* is an MBF target [37], which is transcribed in late G1, and induced by DNA damage and other forms of stress [13]. Msa1 and Msa2 have also been found to form a distinct activatory complex with Ste12 and Tec1 on the *FLO11* and *MSB1* promoters [38]. These genes are involved in cell adhesion and pseudohyphal development and the *msa1msa2* double mutant is adhesion-defective. This led the authors to conclude that the Msa proteins may play a role in coordinating cell division with development.

**Fig 3:**
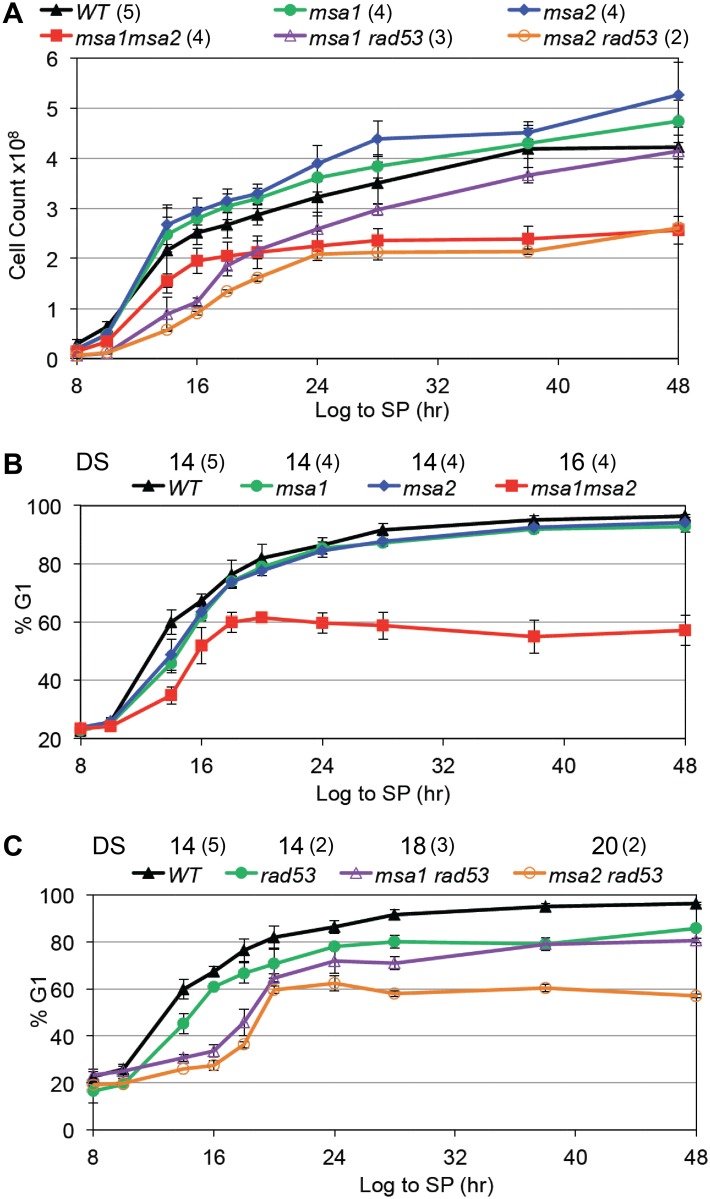
Msa1, Msa2 and the checkpoint function of Rad53 all contribute to the control of proliferation and the G1 arrest that accompanies growth to stationary phase. Cell number (A) and percent of cells in G1 (B and C) are plotted as cells grow from log phase to stationary phase as in Fig 2. The hour after which the diauxic shift (DS) has occurred for each strain is noted above. Genotypes as indicated. Number of trials averaged for these figures is shown in parentheses.

**Fig 4:**
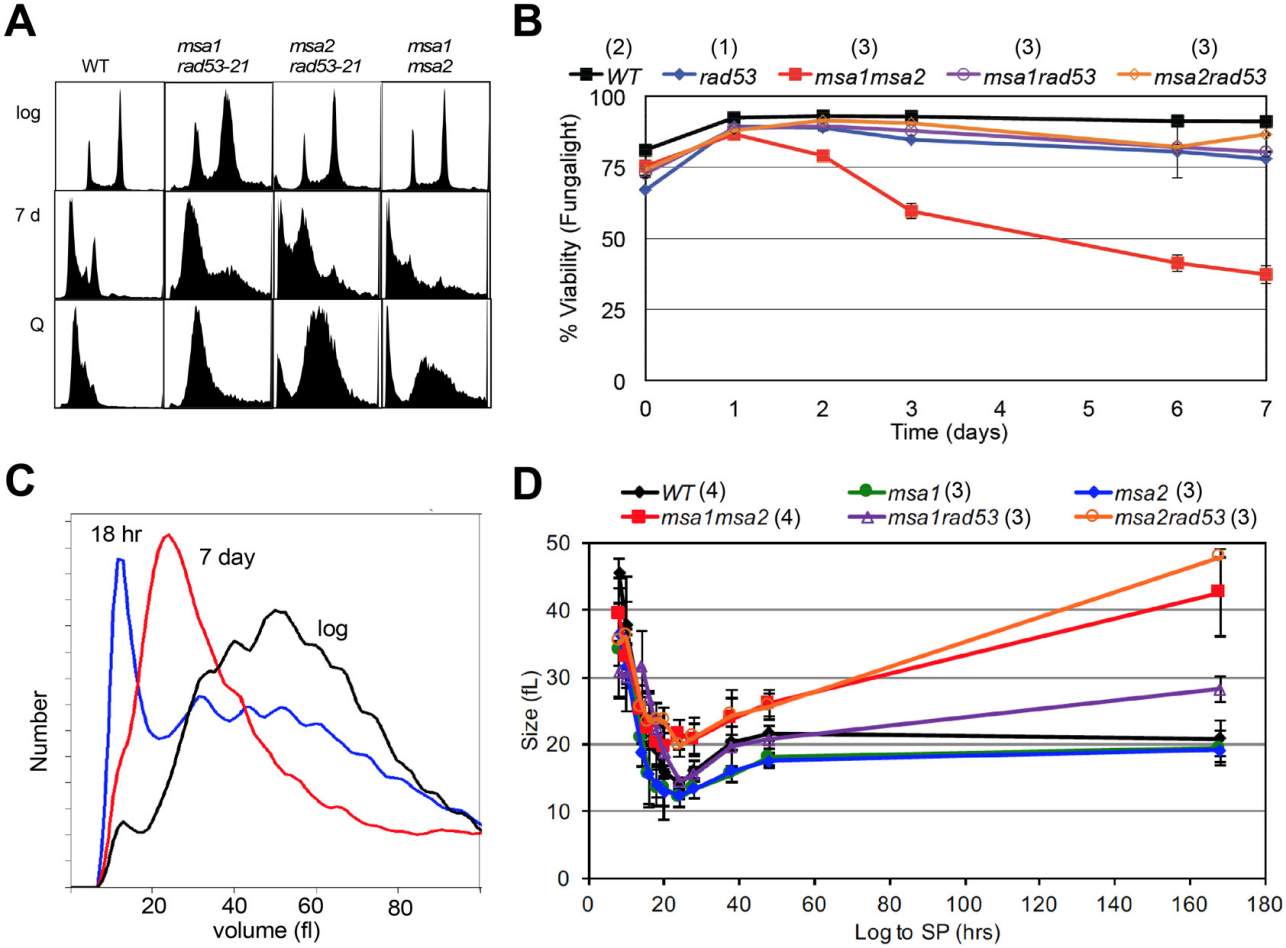
Msa1 and Msa2 and the checkpoint function of Rad53 contribute to the control of cell expansion and survival. (A) Flow cytometry histograms of DNA content of cells during logarithmic growth (log), after seven days of growth to saturation (7d) and from purified quiescent cells (Q). Genotypes as indicated. The two prominent peaks in log phase wild type (WT) cells reflect the 1N and 2N DNA content of cells in G1 and G2/M, respectively. The trough between the peaks reflect cells in S phase. Note that over 90% of WT cells are in G1 after seven days, and the DNA of the cells that purify as Q cells shifts to a sub-1N position. This shift is due, at least in part, to their resistance to dye penetration [41]. Most of the *msa1msa2* cells contain very low and heterogeneous DNA content after seven days (7 d) of growth. This is correlated with loss of viability. (B) Plots of viability over a seven day time course of growth in rich medium for the genotypes indicated. (C) Representative Coulter Counter trace of cell volume distributions of wild type (BY6500) cells in log phase compared to that of cells six hours after the diauxic shift (18 hours), and after seven days of growth. (D) Plot of modal cell volume of the genotypes indicated as they undergo asymmetric cell divisions and enter stationary phase over a seven day time course. Number of trials averaged for these figures is shown in parentheses.

Our data indicate that both Msa1 and Msa2 are critical for cell division arrest and growth arrest as cells transition to quiescence. In prototrophic W303, loss of either Msa1 or Msa2 or both has no effect on the fraction of time the cells spend in G1 when they are growing logarithmically. The fraction of log phase cells that are in G1 in the single and double *msa* mutants is comparable to wild type (Table 1 and S1 Fig). However, as these cultures increase in cell number, the double mutant stops dividing at about half the cell density of wild type cultures (Fig 3A). With wild type cells, the percent of cells in G1 triples as they approach the diauxic shift [14] and Fig 3B). The single *msa* mutants are slightly delayed if at all in this response compared to wild type cells. In contrast, the *msa1msa2* double mutant shows a slow accumulation of G1 cells that plateaus at 60% after 18 hours of growth (Fig 3B). After seven days of growth, the *msa1msa2* cells show very low heterogeneous DNA content (Fig 4A) and most of the cells are dead (Fig 4B). Not surprisingly, the *msa1msa2* Q cell yield is also low (red dot in Fig 1A).

The failure of the double mutant to arrest in G1 and its loss of viability over this time course suggests that Msa1 and Msa2 play redundant roles in halting cell cycle progression specifically during the transition to quiescence. However, in checkpoint-deficient cells, carrying the *rad53-21* mutation [39], Msa1 and Msa2 are both required for efficient G1 arrest in response to nutrient consumption (Fig 3C) We have previously shown that the Rad53-mediated replication stress checkpoint plays a role during the transition to quiescence [14]. Just as in rapidly cycling cells [11, 12, 40], Rad53 function is essential for restraining cells in G1 and achieving quiescence when the transition to S is driven prematurely by excess Cln3 [14] and Table 1). If the Msa proteins are also important for G1 arrest, we expected that their absence would also exacerbate the *rad53-21* phenotype, and this is exactly what we observe. As noted previously [14], checkpoint-deficient *rad53-21* cells do not achieve the full G1 arrest observed with wild type cells after 48 hours of growth (Fig 3C). The additional loss of either Msa1 or Msa2 mutants has a more extreme phenotype. These double mutants are almost as defective in cell cycle arrest after 48 hours of growth as the *msa1msa2* mutant (Fig 3C). This indicates that when Rad53 is not present to reinforce the arrest, Msa1 and Msa2 are both required to efficiently halt cell cycle progression. *msa2rad53-21* has the most extreme defect. It is an unstable strain and we were unable to construct the *msa1msa2rad53* triple mutant. However, in contrast to *msa1msa2*, most of the *msa1rad53-21* and *msa2rad53-21* cells achieve G1 arrest after seven days in culture (Fig 4A) and retain viability (Fig 4B).

### Msa1 and Msa2 also restrain physical growth of cells as they transition to quiescence

**Fig 5:**
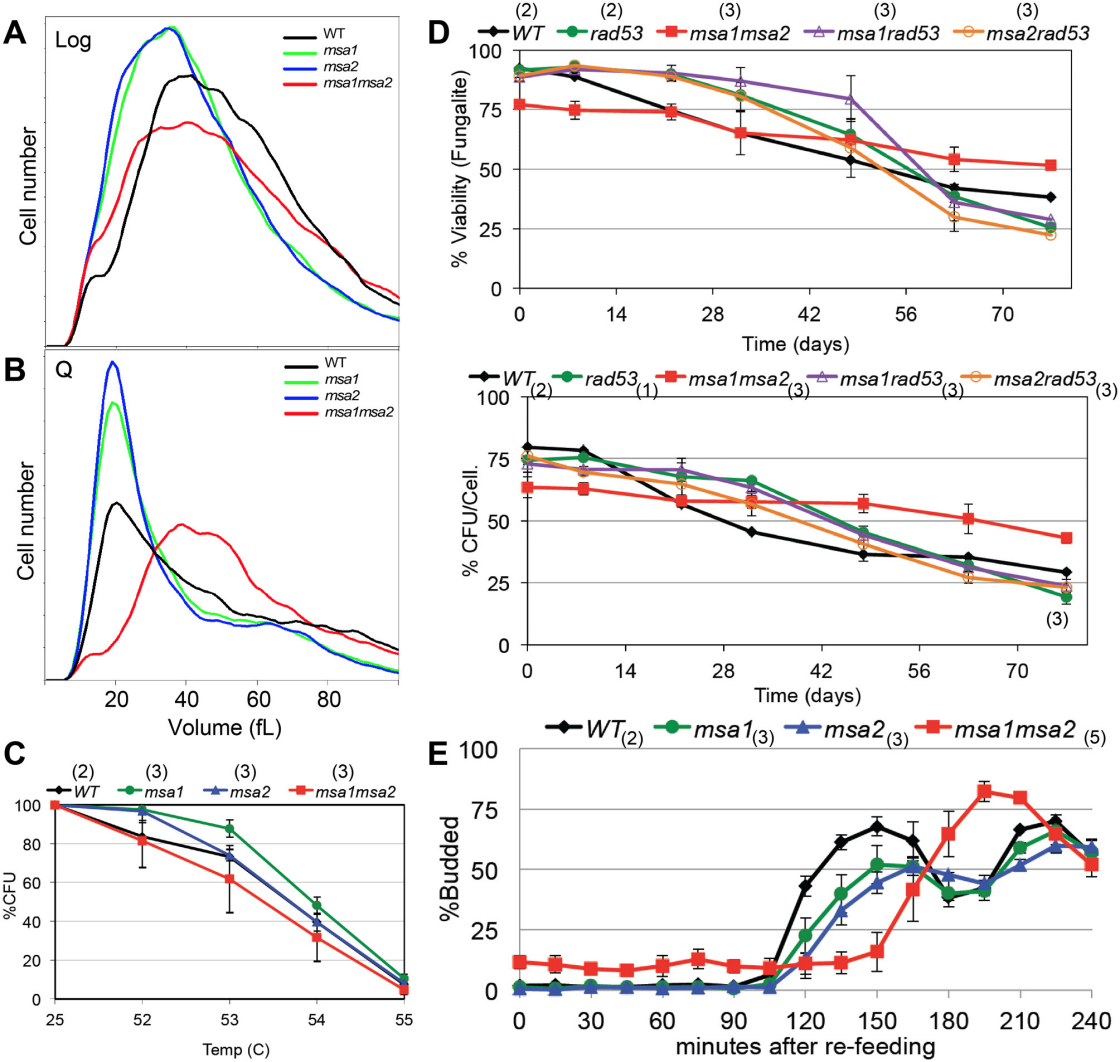
*msa* mutant cells that survive growth to saturation attain many properties of wild type quiescent cells. (A) Cell size of *msa* single and double mutants from log phase cultures are compared to (B) the size of these mutants after growth for seven days and purification of the high density quiescent (Q) cell fraction. One representative Coulter Counter trace is shown for each strain. Those high density Q cells were then assayed for three known properties of wild type quiescent cells: (C) thermo-tolerance, or survival after a ten minute incubation at the temperatures indicated, (D) longevity as assayed in Fig 2, and (E) cell cycle re-entry of purified Q cells as assayed by bud formation after transfer to YEPD medium. Number of trials averaged for these figures is shown in parentheses.

Our previous work shows that shortly after the diauxic shift, wild type cells undergo a dramatic shift in cell size, due to a highly asymmetric cell division [15]. Fig 4C shows the cell size distribution of wild type cells during logarithmic growth compared to that of cells after the diauxic shift (18hr) and after seven days in culture. The asymmetric cell division of wild type cells gives rise to daughters that are about 14 femtoliters (fL) in volume. These cells slowly increase in volume to about 20 fL and never attain the 40–60 fL volume observed in log phase cultures. To see if the Msa proteins are required for this asymmetric cell division, we plotted the modal cell size of the single and double mutants as they grew from log to stationary phase (Fig 4D). All five strains undergo asymmetric cell division, but the *msa2rad53-21* and *msa1msa2* cells continue to enlarge. *msa1rad53-21* has an intermediate phenotype. Interestingly, the *msa2rad53-21* cells increase to the same size as *msa1msa2* cells, but do not lose viability over this time course. They also produce nearly wild type levels of Q cells (Table 1). This is correlated with and may be explained by the fact that the majority of the *msa1rad53* and *msa2rad53* cells eventually attain G1 arrest after seven days of growth (Fig 4A). Despite their large size, the viable cells in G1 purify as Q cells from those seven day cultures (Figs 4A and 5D) We conclude that Msa1 and Msa2 both contribute to efficient cell division arrest and cell growth arrest as nutrients become limiting. Rad53 checkpoint function provides critical backup in restraining cell cycle progression and growth when either Msa protein is absent. However, Msa1 and Msa2 have critical overlapping roles in achieving full G1 arrest and maintaining viability during the transition to quiescence.

### Msa1 and Msa2 are required for the transition to quiescence, but not to the maintenance of that state

To see if the survivors of this transition achieve a protective quiescent state, we purified the high density cells from a seven day old culture of *msa1*, *msa2* and *msa1msa2*. Fig 5A and 5B compare the size of these cells from the starting log phase culture and the purified high density Q cells. Both *msa* mutants are smaller than wild type cells during logarithmic growth, but the *msa1msa2* cells are clearly larger and more heterogeneous than the single mutants. The small size of the single mutants could indicate that they spend less time in G1, but this is not born out by their flow cytometry profiles, which look like wild type (S1 Fig). The *msa1msa2* cells also have a flow cytometry profile very similar to the wild type profile during logarithmic growth (Fig 4A). This suggests that their overlapping function is not important during rapid growth. After seven days of growth to stationary phase, we obtained wild type yields of high-density cells from the *msa1* and *msa2* mutants (61 ±3% and 57 ±2% respectively.) However, the double mutant produces a lower, more variable yield of 34 ±6% (Table 1). The dense *msa1msa2* cells are also very large (Fig 5B), consistent with their inability to cease cell growth (Fig 4D). Despite their large size, one third of the *msa1msa2* cells achieve the density characteristic of Q cells. To see if these high-density cells attained other features of quiescent cells, we tested their thermo-tolerance (Fig 5C) and their longevity (Fig 5D). The dense fraction of *msa1msa2* cells is comprised of 75% viable cells (Fig 5D), compared to only 30% viable cells found in the seven day old cultures (Fig 4B). Interestingly, these high-density cells have the same thermo-tolerance and longevity of wild type quiescent cells (Fig 5C and 5D.) We conclude that Msa1 and Msa2 are critical for the efficient transition into quiescence, but the cells that survive this transition achieve at least some of the protective features of Q cells.

### Efficient cell cycle re-entry also requires Msa1 and Msa2 activity

Another feature of quiescent cells is their rapid and synchronous return to the cell cycle upon re-feeding [20]. Fig 5E shows the typical 90 minute delay, followed by a highly synchronous cell division, that we observe when Q cells are transferred from water to rich media. The single *msa* mutants are clearly delayed, and the *msa1msa2* cells lag considerably longer. The starting population of *msa1msa2* Q cells is about 15% budded, but these budded cells are likely dead, based on their phase dark appearance in the microscope and their failure to progress. The unbudded population begins to bud after 135 minutes. These delays show that both Msa1 and Msa2 are important for an efficient transition out of quiescence, but they are not required for this transition because eventually nearly all the live cells bud.

### Msa1 and Msa2 regulate MBF and SBF targets in post-diauxic cells

**Fig 6:**
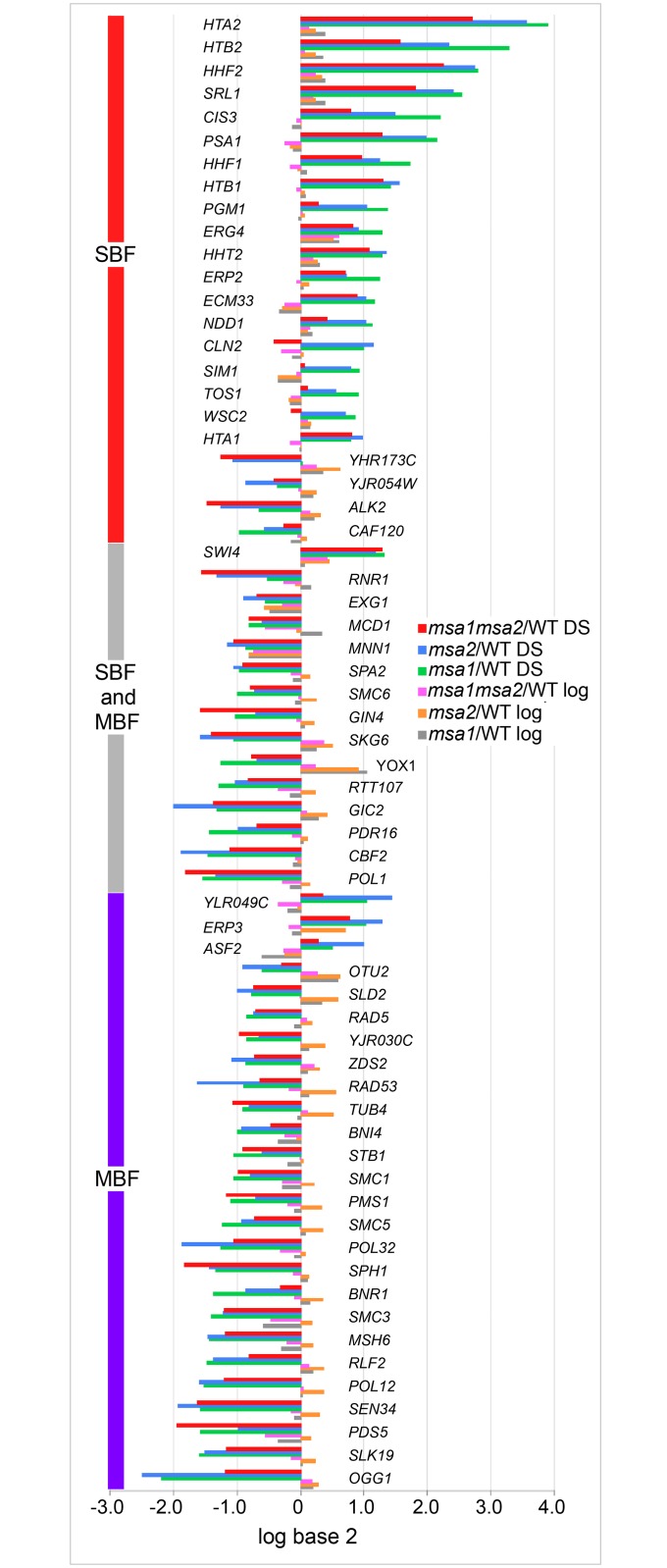
SBF and MBF target mRNAs are differentially regulated by Msa1 and Msa2. RNA deep sequencing was used to quantify mRNA levels genome-wide from wild type and *msa* single and double mutants during the log phase of growth and after the diauxic shift (DS). These mRNA levels are displayed as the log base 2 ratio of mutant over wild type as indicated in the legend. Only transcripts that are affected by log base 2 = .8 or greater (about 1.8-fold) in either *msa1*/WT or *msa2*/WT are shown. Transcripts meeting this criterion and their designations as SBF and/or MBF targets [37] are shown.

The failure of the *msa1msa2* mutant to arrest in G1 and the association of these proteins with both SBF and MBF led us to ask if known MBF and/or SBF targets were deregulated in these mutants as they transition from log phase to quiescence. As discovered previously [18], *msa1* and *msa2* mutants have minimal impact on transcription during the log phase of growth. However, they have a significantly greater influence on transcription after the diauxic shift (S2 Fig). To see if MBF and SBF targets are affected, we looked for known MBF and/or SBF targets [37], which were negatively or positively affected by *msa1*, *msa2* or the double mutant. Using a cutoff of 1.8-fold, we found that about half the known SBF and MBF targets were affected by *msa1* or *msa2*. Fig 6 shows the levels of these MBF and SBF target transcripts in the *msa* single and double mutants in log phase cells and in cells that have undergone the diauxic shift, expressed as a ratio of mutant over wild type. With the exception of *YOX1* and *MNN1*, none of these transcripts are significantly affected by the *msa* mutants during the log phase of growth. However, after the diauxic shift, we find that MBF targets, and targets of both MBF and SBF are primarily under-represented, and those that are only SBF targets are primarily elevated in the mutants. We also find that the impact of *msa1* and *msa2* is similar at these promoters and their effects are not additive. This indicates that both Msa1 and Msa2 are required to regulate these MBF and SBF targets in post-diauxic cells. The fact that loss of both Msa proteins has about the same effect at these targets as loss of either one suggests that both Msa proteins are critical components of the same pathways that confer this regulation. It is also worth noting that while only about half the known targets of these late G1-specific transcription factors meet the 1.8-fold threshold in either single mutant, many others are affected in the same way but to a lesser extent (S1 Table). We predict that other transcriptional regulators, mRNA stability or mRNA sequestration are likely to be variables that complicate the extent of de-regulation we observe.

The set of MBF targets whose activation in post-diauxic cells require both Msa1 and Msa2 include *SLD2* and a number of other genes involved in DNA replication. In fact, 22 of the 54 MBF targets (p value = 10^−9^) most repressed in the *msa* single mutants are involved in DNA metabolism and 18 are involved in DNA repair (p value = 10^−10^.) This may help explain why galactose-induced overproduction of Msa1 suppresses *sld2* and some other DNA replication defects [18]. *SLD2* is also an *MSA2* activated gene, but Msa2 over-expression does not suppress *sld2*. This asymmetry could be explained if *MSA2*, itself an MBF target, is also over-expressed upon galactose-induction of Msa1. This would result in high levels of both Msa proteins, which would activate *SLD2* and other DNA replication genes. In contrast, high levels of Msa2 would not be expected to induce high levels of Msa1, and since both are required for activation of these replication genes, high Msa2 alone would not have the same suppressing effect.

The next most enriched class of MBF targets that are activated by both Msa1 and Msa2 are genes involved in sister chromatid cohesion (*SMC1*, *SMC3*, *IRR1*, *PDS5*, *MCD1* and *CSM3*.) It is known that genome-wide cohesion occurs in response to a single double strand break [42, 43]. Moreover, this break-induced cohesion prevents the loss of the unbroken chromosomes, indicating that it serves a purpose beyond repair of the single double strand break [43]. The co-activation of cohesion genes as cells enter quiescence brings up the intriguing possibility that cohesion may protect and/or compact the genome in quiescent cells. It has recently been shown that quiescent cells have uniquely compact chromatin in which all the telomeres are in a tight cluster in the center of the nucleus [44, 45].

At the SBF target promoters, where both Msa1 and Msa2 are required for the repression, the histone transcripts are among the most affected. All eight core histone transcripts are highly elevated in the *msa* mutants (Fig 6 and S1 Table). *HHT1* is not pictured in Fig 6 because it missed the cutoff for being an assigned SBF target [37]. In addition, the linker histone *HHO1* and the H2A variant *HTZ1*, both barely missed our cutoff for inclusion on Fig 6, each being about 1.7-fold elevated above wild type in post-diauxic *msa2* cells. There are 29 targets that meet or exceed the 1.7-fold mRNA level increase and 10 of them are histones. The only other histone, *CSE4*, which is centromere-specific, is unaffected. Histone expression is tightly controlled by multiple mechanisms that are not entirely understood despite decades of investigation [46]. To confirm the role of SBF in transcription of these genes, we assayed the *HTA1* promoter activity through the cell cycle in *swi4*, *mbp1* and *swi4mbp1* cells. S3 Fig shows that Swi4 contributes to the cell cycle-specific activation of this promoter and Mbp1 has much less effect.

Our data indicate that Msa1 and Msa2 both make independent contributions to the post-diauxic regulation of many MBF and SBF targets. Loss of these activities is not additive, suggesting that they participate in the same pathways of regulation at these promoters. However, the extreme phenotype is only observed in the double mutant. This suggests some redundancy of function. Redundancy is also suggested by the sequence similarity between Msa1 and Msa2. To address this question, we looked across the genome for transcripts that were mis-regulated in the *msa1msa2* double mutant, but not mis-regulated to the same extent in either single mutant in the post-diauxic time point. Table 2 lists all the transcripts across the genome that are mis-regulated 1.7 fold or more in *msa1msa2* but less so in *msa1* or *msa2* after the diauxic shift. Most of these transcripts also show additivity, in that the single mutants mis-regulate in the same way, but to a lesser extent. This can be readily seen in the bar graph in S4 Fig. What is striking from this genome-wide survey is that of the 47 transcripts most down-regulated in the double mutant, one-quarter are already known MBF and/or SBF targets (Table 2). This further supports the view that regulating MBF and SBF activities are the critical functions of Msa1 and Msa2 in post-diauxic cells. Overall, eleven of the 47 genes are cell cycle genes, and all but three of these are involved in chromosome segregation and/or the establishment of polarity in the cell division process. Others affect a diversity of processes.

**Table 2:** Transcripts that require both Msa1 and Msa2 for full activation.

gene	MBF/SBF	peak	description	*msa1*	*msa2*	*msa1*
				/WT	/WT	*msa2*
						/WT
*TOS6*	SBF	26	cell wall protein; decreases in response to ergosterol perturbation and stationary phase	0.43	0.09	-2.03
*CSI2*	SBF	21	unknown function; localizes to the mother side of the bud neck and vacuole	-0.57	0.13	-1.63
*PEA2*^1^^,^^3^		20	polarisome subunit; required for polarity establishment	0.01	-0.40	-1.18
***PGA2***			involved in protein trafficking; required for cell separation	0.03	0.24	-1.17
***CDC21***	MBF	20	Thymidylate synthase; involved in synthesis of pyrimidine deoxyribonucleotides	0.00	-0.29	-1.10
*AXL2*^1^^,^^3^	MBF/SBF	21	membrane protein at incipient bud site and bud neck; required for axial budding	0.20	0.33	-1.10
*DYN1*^1^^,^^2^^,^^3^			Cytoplasmic dynein; required for spindle assembly and chromosome movement	-0.29	-0.26	-1.10
*FHN1*			unknown function; promoter contains sterol regulatory element	-0.02	-0.01	-1.07
*CPT1*			phosphatidylcholine biosynthesis and inositol-dependent EPT1 transcription;	-0.37	-0.46	-1.06
*CLN1*^1^	MBF/SBF	21	G1 cyclin; activates Cdc28p kinase to promote the G1 to S phase transition	-0.40	-0.08	-0.98
*PRY2*	SBF	24	Sterol binding protein involved in the export of acetylated sterols	0.38	-0.18	-0.95
*YBL111C*			Helicase at telomeric Y' element; moves to cytoplasm upon DNA replication stress	-0.41	-0.25	-0.95
*YOR114W*	MBF/SBF	21	Putative protein of unknown function; null mutant is viable	-0.27	-0.49	-0.94
*RKM1*	MBF	20	SET-domain lysine-N-methyltransferase; methylates ribosomal proteins	-0.38	-0.59	-0.93
*ADK2*		16	Mitochondrial adenylate kinase; catalyzes synthesis of GTP and AMP	-0.46	-0.35	-0.87
*YMR144W*	MBF/SBF	34	unknown function; localized to the nucleus; YMR144W is not an essential gene	0.14	-0.35	-0.86
*SCP1*			yeast cortical actin cytoskeleton protein; cross links actin filaments	-0.13	-0.47	-0.85
*SET5*			Methyltransferase involved in methylation of histone H4 Lys5, -8, -12	-0.12	-0.48	-0.85
*PEX14*			peroxisomal importomer complex component	-0.55	-0.49	-0.85
*CTF19*^1^^,^^2^			kinetochore protein, chromosome segregation and spindle assembly checkpoint	-0.54	-0.18	-0.84
*OPT2*			Oligopeptide transporter; affects vacuole formation and polarized cell growth	-0.46	-0.38	-0.83
***MDM1***			Intermediate filament; nuclear and mt transmission to daughter buds	-0.01	-0.34	-0.83
*YJL147C*			Mitochondrial protein of unknown function; required for sporulation	-0.32	-0.56	-0.82
*YMR147W*			Putative protein of unknown function	0.41	0.10	-0.81
*DCI1*			Peroxisomal protein; may be involved in fatty acid metabolism	-0.57	-0.42	-0.81
*PDR8*			Transcription factor; targets genes involved in the pleiotropic drug resistance	-0.51	-0.06	-0.81
*RNH201*		19	Ribonuclease H2 subunit; removes RNA primers during DNA replication	-0.17	0.07	-0.81
*SVS1*	SBF	25	Cell wall and vacuolar protein; required for wild-type resistance to vanadate	0.57	0.42	-0.81
*BST1*		35	GPI inositol deacylase; discriminates between ER and Golgi-bound molecules	-0.42	-0.60	-0.79
*KRE6*			beta-1,6 glucan biosynthesis; in ER, plasma membrane, sites of polarized growth	0.03	-0.47	-0.79
*TSC3*			involved in sphingolipid biosynthesis	-0.21	-0.60	-0.78
*YIL102C-A*			Putative protein of unknown function	-0.59	-0.50	-0.78
***DAD1***^1^^,^^2^			DASH complex subunit of kinetochore; involved in chromosome segregation	-0.32	0.03	-0.76
*ACM1*^1^	MBF/SBF	15	inhibitor of the APC/C [Cdh1]-mediated proteolysis of mitotic cyclins	-0.22	0.49	-0.76
*BUD13*^1^^,^^3^			pre-mRNA splicing; MATa1 splicing defect confers haploid expression in diploids	-0.20	-0.36	-0.75
*IRC15*^1^^,^^2^			Microtubule associated; meiotic chromosome segregation and mitotic progression	-0.46	-0.11	-0.75
***ORC4***^1^			origin recognition complex subunit; DNA replication and transcriptional silencing	-0.28	-0.34	-0.75
*PDS1*^1^^,^^2^	MBF/SBF	32	Securin; inhibits anaphase; blocks cyclin destruction and mitotic exit	0.34	-0.08	-0.74
***AAR2***			U5 snRNP complex; required for splicing of U3 precursors	-0.46	-0.20	-0.73
*ERP4*			involved in ER to Golgi transport	-0.16	-0.41	-0.73
*DDI3*			Protein of unknown function; expression is induced over 100-fold by DNA damage	-0.32	-0.32	-0.73
*CCC1*			Vacuolar Fe2+/Mn2+ transporter; leaves vacuole upon DNA replication stress	-0.56	-0.48	-0.72
***POP5***			RNase MRP and RNase P subunit; generates mature 5' ends of nuclear RNAs	-0.21	-0.36	-0.72
*BAP2*			amino acid permease involved in uptake of leucine, isoleucine and valine	-0.42	-0.42	-0.71
*LRP1*			exosome-associated protein; involved in RNA processing, degradation, and export	-0.35	0.21	-0.71
*PFA5*			Palmitoyltransferase; likely functions in pathway(s) outside Ras	-0.38	-0.45	-0.71
*PHM8*		85	Lysophosphatidic acid phosphatase, nucleotidase working on GMP, UMP and CMP	-0.42	-0.42	-0.70

Known SBF and/or MBF targets are indicated in column 2 [37]. Peak times of known cell cycle regulated transcripts are indicated in column 3 [47]. Descriptions are abbreviated from *Saccharomyces* Genome Database. Transcripts levels are shown as log_2_ ratio of mutant over wild type (WT) for the post-diauxic time point. Genes marked with bold text are essential genes. Gene ontology terms are superscripted as:

^1^ cell cycle,

^2^ chromosome segregation,

^3^ establishment of polarity

There is a smaller set of transcripts that are elevated specifically in the double mutant (Table 3). Here again, the single mutants typically also elevate the transcript levels, but to a lesser extent. None of these transcripts are known MBF and/or SBF targets, nor do they show significant enrichment for cell cycle regulation. Rather, seven of these 30 genes respond to stress, and four are meiosis-specific. Another four genes (TIR1, 3,and 4 and DAN1) are specifically expressed during, and required for anaerobic growth [48, 49]. Three of the most elevated transcripts are involved in pyrimidine biosynthesis. There is no reason to think that these are direct targets of Msa1 or Msa2. Rather we suspect that failure to properly initiate the transition to quiescence indirectly results in the ectopic expression of genes involved in other developmental pathways. This ectopic expression could contribute, indirectly, to the loss of viability of the double mutant.

**Table 3:** Transcripts that require both Msa1 and Msa2 for full repression.

gene	MBF/SBF	peak	description	*msa1*/WT	*msa2*/WT	*msa1msa2*/WT
*THI4*^4^^,^^7^			thiamine synthesis; required for mt genome stability in response to DNA damage	0.18	-0.14	1.77
*MAM1*^5^			Monopolin; kinetochore protein; chromosome attachment to meiotic spindle	0.16	0.06	1.75
*YFL012W*			unknown function; null mutant increases resistance to rapamycin	0.42	0.32	1.50
*FUR1*^7^			synthesizes UMP from uracil; involved in the pyrimidine salvage pathway	0.30	0.32	1.27
*YLR162W*			unknown function; overexpression causes growth arrest and apoptosis	-0.58	0.11	1.23
*URA2*^7^			first two enzymatic steps in pyrimidine biosynthesis; feedback inhibited by UTP	0.38	0.12	1.23
*TIR1*^4^^,^^6^		52	Cell wall protein; downregulated in acid, induced by cold shock and anaerobiosis	0.03	-0.12	1.18
*FUI1*			uridine permease; moves to the vacuole upon DNA replication stress	0.42	0.54	1.17
*CRS5*			Copper-binding metallothionein; required for wild-type copper resistance	0.54	0.56	1.17
*YLR194C*		5	cell wall protein; expression is upregulated in response to cell wall stress	0.56	0.29	1.01
*STE3*			a factor receptor; mediates pheromone response; required for mating	0.21	-0.41	0.95
*RPS22B*			Protein component of the small (40S) ribosomal subunit	0.45	0.37	0.92
*ACA1*			ATF/CREB transcription factor; important for carbon source utilization	0.31	0.51	0.89
*DAN1*^4^^,^^6^			Cell wall mannoprotein; expressed only under anaerobic conditions	0.25	-0.52	0.86
*IRC7*			Beta-lyase involved in thiol production; null mutant increases Rad52p foci	-0.31	-0.47	0.84
*SFG1*		66	transcription factor; required for growth of pseudohyphae	0.59	-0.17	0.84
*FDH1*			NAD(+)-dependent formate dehydrogenase	-0.35	-0.18	0.83
*RPL9B*			Ribosomal 60S subunit protein L9B	0.30	0.04	0.81
*AFT1*^4^^,^^5^			Transcription factor for iron deficiency; nuclear upon DNA replication stress	0.24	0.32	0.81
*HSP31*^4^			stress, diauxic shift, and stationary phase survival; up in DNA replication stress	0.24	0.36	0.80
*MEK1*^5^			Meiosis-specific protein kinase; meiotic checkpoint and recombination	0.42	0.32	0.80
*ECM12*			unknown function; mutants display zymolyase hypersensitivity	0.22	0.44	0.77
*YKE4*			Zinc transporter in the ER; null mutant leads to zinc accumulation in cytosol	0.26	0.38	0.76
*SNA2*			unknown function; has similarity to Pmp3p, which is involved in cation transport	-0.12	0.47	0.76
*YJL213W*			unknown function that may interact with ribosomes	0.05	-0.04	0.75
*TIR3*^4^^,^^6^		65	Cell wall mannoprotein; required for anaerobic growth	0.60	0.26	0.74
*SMF3*			metal transporter involved in iron homeostasis; increases in DNA replication stress	0.38	0.52	0.73
*DAL4*			Allantoin permease; expression sensitive to nitrogen catabolite repression	0.01	0.36	0.73
*TIR4*^4^^,^^6^			Cell wall mannoprotein; required for anaerobic growth; induced by cold shock	0.04	0.09	0.71
*SPR28*^5^			Sporulation-specific homolog of the Cdc3/10/11/12 family of septins	0.03	0.14	0.71

Columns as reported in Table 2. Gene ontology terms are superscripted as:

^4^ response to stress,

^5^ meiotic cell cycle,

^6^ cell wall protein family,

^7^ pyrimidine biosynthesis.

It is unclear which of the mis-regulated transcripts listed in Table 2 and/or 3 might be responsible for the loss of viability observed in the double mutant. Indeed, there is no reason to assume that a single member of either class is causing loss of viability in the *msa1msa2* mutant. For example, seven of the down-regulated genes are essential for viability (bold in Table 2) and the reduced expression of any or all of these genes could be deleterious. Included among the up-regulated transcripts is an uncharacterized gene (YLR162W), which is known to cause growth arrest and apoptosis when over-expressed [50].

### Msa1 and Msa2 bind directly and independently to their targets

**Fig 7:**
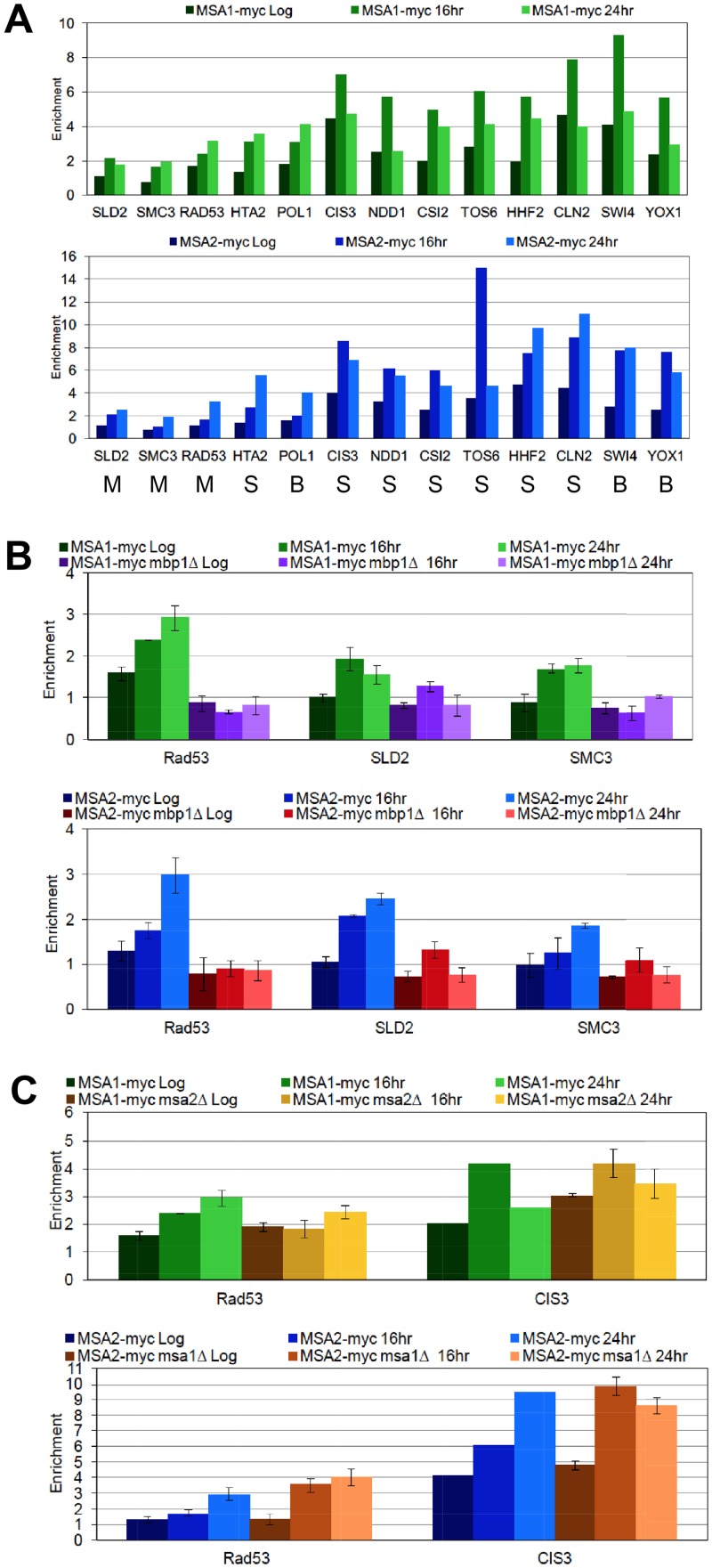
Msa1 and Msa2 bind both SBF and MBF target genes. Chromatin immunoprecipitation (ChIP) assays were carried out to detect *in vivo* binding of Msa1 (top panels) or Msa2 (bottom panels) under different conditions. (A) Binding of myc-tagged Msa proteins was surveyed at 13 potential target promoters at three time points: during log phase (Log), and after the DS (16 hr) and (24hr.) Whether they are targets of SBF (S), MBF (M), or both (B) is indicated below. (B) Three MBF targets were re-assayed in triplicate. These show relatively weak binding, so the ChIP analysis of Msa1 and Msa2 binding to these three MBF targets was repeated in the absence of Mbp1, confirming that the binding is Mbp1-dependent. (C) Neither binding of Msa1 or Msa2 to an MBF target (*RAD53*) or an SBF target (*CIS3*) is dependent upon the other. Msa1 binding was observed in the presence or absence of Msa2, and Msa2 binding was observed in the presence or absence of Msa1. These assays are carried out in triplicate with the following strains: BY7388 *MSA1-myc-KanMx*, BY7459 *MSA1-myc-KanMx msa2*::*HIS3*, BY7390 *MSA2-myc-KanMx*, BY7461 *MSA2-myc-KanMx msa1*::*HIS3*, *BY7561 MSA1-myc-KanMx*, *mbp1*::*HIS3*, and BY7562 *MSA2-myc-KanMx*, *mbp1*::*HIS3*.

The down-regulation of MBF targets in the absence of Msa activity could be due to direct binding and activation by the Msa/MBF complex, or it could be due to indirect effects on other regulators. We note that *NRM1* is slightly elevated and *RAD53* is substantially down-regulated in the *msa* mutants (S1 Table). Rad53 is known to phosphorylate and release the negative regulator Nrm1 from MBF complexes in the presence of DNA damage [13, 51]. Similarly, the high levels of SBF target transcripts in the *msa* mutants may be due to direct repression by the Msa/SBF complex, or it could reflect indirect effects on other regulators that are mis-expressed in the Msa mutants. For example, Swi4, the DNA binding component of SBF is up-regulated in the *msa* mutants (Fig 6). To see if Msa regulation of these targets is direct or indirect, we assayed binding of Msa1 and Msa2 to a set of SBF and MBF targets by chromatin immunoprecipitation. Fig 7A shows a survey of Msa binding to thirteen of these genes, including eleven from Fig 6 and two from Table 2 (*TOS6* and *CSI2*.) We see robust binding signals for both Msa proteins on SBF targets (S) and on promoters containing both SBF and MBF binding sites (B). Binding is weaker to the MBF targets (M). In most cases, the binding signals are higher in the post-diauxic time points (16 and 24 hours.) To confirm the relatively weak binding to MBF targets, we asked if the binding was dependent upon Mbp1, which is the DNA binding component of MBF [9]. Fig 7B shows that the binding observed at three MBF target promoters in post-diauxic cells is Mbp1-dependent. Fig 7C shows that the binding of Msa1 does not depend on the presence of Msa2, or vice versa, at both MBF and SBF targets. These data are most consistent with the direct binding of Msa1 and Msa2 to both classes of late G1-specific promoters. The Msa-dependent regulation of these transcripts is likely to be important for preventing entry into S phase as cells respond to a waning nutrient supply and enter quiescence.

## Discussion

The transition from proliferation to quiescence involves a stable but reversible cell cycle arrest in G1. It follows that the transcriptional regulators that drive the G1 to S transition have to either be eliminated or reprogrammed. Perhaps because of the need for rapid reversibility of this arrest, budding yeast utilize their E2F-like complex of Swi4 and Swi6 (SBF) as a platform for a novel form of regulation that involves Msa1 and Msa2 and is initiated as glucose levels drop. The regulation conferred by Msa1 and Msa2 is critical for the cell cycle arrest, cell growth arrest and viability of cells as they transition to quiescence. Other known SBF regulators (Stb1, Whi5, Srl3) do not play a significant role in the transition into quiescence, but Whi5 and the related Srl3 protein seem to accelerate the reversal of quiescence when nutritional conditions improve. This is opposite their roles as negative regulators in exponentially growing cells [6, 7, 29]. One possible explanation is that Whi5 (and/or Srl3) may displace the Msa proteins from SBF as an early step in the recovery phase. Such an exchange would maintain repression of SBF targets, but would make their activation responsive to the increased cyclin levels that accompany the transition to S phase.

The Msa1 and Msa2 proteins were identified by two very different strategies. The MudPIT analysis used to identify Whi5 and Nrm1 as components of the SBF and MBF complexes [7] also identified two related proteins that were named MBF- and SBF- associated (Msa) proteins [17]. Msa2 had been shown to interact with Swi6, the common component of SBF and MBF [52], and both *MSA* genes were known to be transcribed in a cell cycle-specific manner. *MSA1* (YOR066W) is transcribed at the M/G1 boundary in a Yox1/Mcm1-dependent manner [34, 53], and *MSA2* is a late G1-specific transcript [54]. Both proteins are also expressed only in G1 and they undergo cell cycle-specific modifications in growing cells [17, 18]. Msa1 is among the handful of proteins whose nuclear localization is regulated by cyclin-dependent kinase activity and is G1-specific, like Whi5 [55]. Both proteins bind to SBF and MBF target promoters, specifically during G1 in cycling cells, and that binding is Swi4- and Mbp1-dependent, respectively [17].

Msa1 was also identified as a high copy suppressor of three temperature sensitive DNA replication mutants: *drc1-1*/*sld2*, *dbp11-1* and *pol2-12* [18]. Interestingly, Msa1 over-expression had a deleterious effect on other DNA replication genes (*cdc6-1* and *cdc7-1*) and other cell cycle regulators (*cdc28* and *cdc14-1*.) Being aware of the previous study showing the interaction of Msa1 with the SBF and MBF transcription complexes [17], these authors carried out genome-wide chromatin immunoprecipitations and transcript microarrays to identify Msa1 targets. They found 50 genes that were both bound and regulated by Msa1 in cycling cells. These genes affect all aspects of cell growth, but showed no clear connection to DNA replication. This left the mystery of Msa1’s role in DNA replication unresolved. They did identify about 60 MBF and/or SBF targets as binding sites for Msa1, but most were not Msa1 regulated in cycling cells, just as we observe. Our study has shown that there are many DNA replication genes that are activated by Msa proteins, including *SLD2*, that could explain the suppression of DNA replication defects, but this only occurs in post-diauxic cells.

One thing that these studies, as well as the study implicating Msa1 in osmoregulation of the cell cycle [19], have in common is the relatively mild phenotypes observed for the *msa* single and double mutants in cycling cells. Altering the levels of Msa1 causes modest changes in the timing of late G1-specific transcription [17], and the initiation of S phase [18]. Further, no synergistic effects in the *msa1msa2* double mutant were reported in cycling cells. We observe similarly mild phenotypes for these mutants during the log phase of growth. This is surprising, considering the many layers of regulation that are exerted upon these proteins in cycling cells, and the critical roles their putative targets play in the G1 to S transition. However, we observe strong deleterious effects of these mutants after the diauxic shift, when cells are preparing to shift from proliferation to quiescence. The Msa proteins are critical during this transition, and one clear effect that they have is in the reprogramming of SBF and MBF activity. Our data suggest that this reprogramming is important for entry into and recovery from quiescence.

We know that the Msa proteins are produced, localized to the nucleus and have the capacity to bind SBF and MBF targets specifically during the G1 phase of every cell cycle. However, only after the cells receive a signal of nutrient limitation are the Msa proteins able to influence the activity of most of these transcription complexes. We propose that the purpose of this tight G1-specific regulation in cycling cells is to ensure that these proteins are present, in the G1 nucleus, to respond immediately to these environmental signal(s), to modulate late G1 transcription and to promote G1 arrest and cell growth arrest. In this way, cells in other phases of the cell cycle would continue to progress and only the G1 cells would initiate cell cycle arrest. This may also explain why there is a correlation between the length of G1 in cycling cells and the ability to enter the quiescent state (Fig 1A).

The majority of the Msa1- and Msa2-dependent regulation we observe fits into one of two patterns. In many cases, loss of either Msa1 or Msa2 disrupts regulation, and loss of both is not additive, indicating that both Msa proteins are required in the same pathway of regulation. In other cases, we see maximum deregulation in the double mutant, which suggests some redundancy. However, in most of these cases, both of the single mutants also de-regulate but to a lesser extent. Though there are exceptions (Tables 2 and 3), the bulk of the evidence suggests that both Msa proteins are required at most promoters. Consistent with this, if we eliminate Rad53 checkpoint function, we see deleterious effects of the single *msa1* or *msa2* mutants that are qualitatively similar but less severe than that of the *msa1msa2* mutant. We conclude that Msa1 and Msa2 have undergone substantial functional divergence, but there is a set of critical targets at which either Msa protein can regulate to a sufficient extent to promote survival during the transition to quiescence.

We have shown that Mbp1 is required for Msa binding at three MBF target sites, but deletion of Mbp1 does not interfere with the transition to quiescence (Fig 1B and Table 1). In contrast, the principle SBF components: Swi4 and Swi6, are required for a normal transition to quiescence. This makes it most likely that the critical targets of Msa regulation that promote G1 arrest are among the SBF targets that they regulate. However, more complicated scenarios are possible. Swi4 and Mbp1 have similar DNA binding domains and similar binding sites [2], and there are instances in which an SBF binding site in G1 becomes an MBF binding site in S phase [56]. These and other complexities make it difficult to guess which of these transcripts could play a critical role in promoting a stable G1 arrest and thereby be responsible for the loss of viability of the double mutant. Further work will be required to determine how Msa1 and Msa2 activity is modulated by nutritional cues, how they achieve this regulation and which of their direct or indirect targets are responsible for the G1 arrest that occurs as cells transition to quiescence.

Despite the lack of physical similarity at the protein sequence level, there are striking parallels between the transcriptional regulation that promotes the G1 to S transition in yeast and mammalian cells [2]. Like SBF and MBF, there are E2F protein complexes that activate transcription in G1 and promote S phase. These complexes are inactive in early G1 due to the binding of repressors (Whi5 and RB), which recruit histone deacetylases to their target genes. In both cases, activation requires removal of the repressors by cyclin-dependent kinases. This enables them to induce transcription of their target genes, many of which are also conserved (e.g. cyclins, replication proteins and histones.) With this work, we extend this conservation of strategy to the transcriptional regulation that promotes the transition from G1 to quiescence. In higher eukaryotes, entry into quiescence depends on the formation of novel E2F complexes that serve to repress these same target genes [4]. This so-called DREAM complex of DP, RB-like, E2F and MuvB was first identified in worms and flies [3, 57] and later found to perform a similar function in human cells [4]. It is assembled on E2F target genes to repress transcription in cells entering quiescence and disruption of these complexes drives cells back into the cell cycle [5]. Msa1 and Msa2 perform a similar function, by binding SBF and MBF complexes and reprogramming their activities. They are not required in cycling cells, but they are critical for the transition to quiescence. In their absence, cells fail to arrest in G1 and lose viability. Interestingly, Msa1and Msa2 do not significantly affect the longevity of cells that successfully enter quiescence, but they are required for efficient entry to and exit from quiescence. It will be interesting to determine how cell cycle re-entry from quiescence differs from the G1 to S transition in cycling cells and which of their targets are rate limiting for this transition.

## Materials and Methods

### Strain construction

All yeast strains used in this study are isogenic with BY6500, the prototrophic version of W303 [58], unless otherwise indicated. Strain numbers are provided in Table 1 or in figure legends. The *5xCLN3* was created by integrating four additional copies of *CLN3* at different marker loci [14] with the integrating vectors, pRS303-306 [59]. The W303 *SSD1* was created as described [58]. All the deletions were made using the Longtine deletion vectors [60] unless otherwise indicated. The checkpoint deficient *rad53-21* mutant [39] was crossed with the prototrophic W303 (BY6500) and then crossed with the deletion strains as listed in Table 1. The myc-tagged strains were constructed using pFA6a-13Myc-KanMX6 [60].

### Growth assays

Viability was monitored by Live/Dead FungaLight (Invitrogen, Grand Island, NY) and colony formation as described. Cell size and cell number was measured on a Z2 Beckman Coulter Counter (Beckman Coulter, Brea, CA.) Growth assays were all carried out in triplicate at 30°C in rich media with 2% glucose (YEPD) with 200 rpm aeration on platform shakers. Growth from log to stationary phase (log to SP) was followed by starting two equivalent 25 ml cultures at an OD_600_ of .02, ten hours apart, from the same culture maintained in log phase. The first culture is sampled at 8, 10, 12, 24, 28 and 48 hours. The second culture is used for the 14, 16, 18, 20 and 38 hour time points. At each time point, samples for cell count, cell size, and flow cytometry were taken. The diauxic shift was determined by the absence of glucose in the media. Glucose levels were determined using glucose detection strips (GLU 300, Precision Labs, Inc. West Chester, OH.) To follow seven days of growth, cultures were inoculated as above. Samples were taken for the zero time point 5 hours after inoculation, then daily, for assaying cell number, viability and colony forming units. Number of trials averaged for these figures is shown in parentheses.

### Quiescent cell assays

Quiescent cells were purified after seven days of growth as described above, by centrifugation through a percoll gradient [15, 20]. Typically 200 OD_600_ units of cells are loaded onto a 25 ml gradient and Q yield is calculated as the percentage of OD_600_ units that sediment to the bottom 9 ml of the 25 ml gradient. The high density Q cells are washed and maintained in water. Longevity of the Q cells was monitored in triplicate from 13 ml suspensions of Q cells in water inoculated to an OD_600_ of 1.0 and incubated with aeration at 30°C. We see no acidification of the water after 300 days of incubation under these conditions with wild type cells. To monitor longevity in the non-dividing state, samples are taken from these Q cell suspensions at two week intervals for cell count, cell size, cell viability and colony forming units. Before sampling, these suspensions are weighed and water is added to replace loss due to evaporation. Thermo-tolerance of the high density Q cell fraction was assayed in triplicate starting with Q cells in water at an OD_600_ of 1.0. 50μl of these cells were transferred to a .5 ml PCR tube and incubated for 10 minutes at the specified temperature. These were chilled, diluted and plated for colony forming units. Q cell re-entry into the cell cycle was followed in triplicate by transferring 10 OD_600_ units of Q cells in one ml into 25 ml of YEPD, sampling at 15 minute intervals and counting percent of budded cells from a total of 200 cells for each time point. Number of trials averaged for these figures is shown in parentheses.

### Flow cytometry

Flow cytometry was carried out as in [15]. DNA content was quantified by staining with Sytox Green and the percent of cells in G1 was determined using the cell cycle module of FlowJo V9.6.4. As we have shown [15], cells transitioning to the quiescent state undergo asymmetric cell divisions and fortify their cell walls. These events give rise to heterogeneity in the flow cytometry profile. In particular, the G1 peak splits into three peaks, which must be added together to obtain the total number of cells in G1 (Fig 4A and S1 Fig). Our plots all report the percent of live cells that are in G1. Dead cells and cell debris, which accumulate in *swi4*, *swi6*, *msa1msa2*, and *msa2rad53* cultures pile up on the left margin of the DNA fluorescence histograms. Number of trials averaged for these figures is shown in parentheses.

### RNA sequencing

RNA sampling, collection and paired end Next-Generation RNA sequencing was carried out as described [14]. mRNA expression levels following polyA selection were assayed by using the HiSeq 2500 next generation sequencing system from Illumina [61] in the Fred Hutchinson Cancer Research Center Genomics Core Facility. Sequences were aligned to the reference genome W303 using the Tophat2 application [62], then counted with HTSeq [63]. Differential expression between samples was measured using the DESeq package from Bioconductor [64]. Ratios of expression in mutant versus wild type were then computed from the normalized read counts. Two biological replicates were generated and averaged for this analysis. These data for all SBF and MBF targets is provided as (S1 Table).

### Chromatin immunoprecipitation

Cells carrying Msa1 or Msa2 tagged with the myc epitope or non-tagged controls were collected from log phase cells, or from cells that had passed the diauxic shift as indicated by the lack of glucose in the media. Proteins were cross-linked to DNA as described [65]. Frozen cell pellets were resuspended in lysis buffer (50 mM HEPES pH 7.6, 140 mM NaCl, 1% Triton X-100, 0.1% Na deoxycholate, 1mM EDTA, 1 mM PMSF, 1 μg/ml aprotinin, leupeptin, and pepstatin A). Cells were broken with glass beads in a Mini Beadbeater-8 (BioSpec Products, Bartlesville, OK) three times for 30 seconds on the Homogenize setting. After a 15-min centrifugation the supernatant was discarded and the pellet (chromatin fraction) was resuspended in the initial volume of lysis buffer. The DNA was fragmented to ∼500 base pairs with a Sonifier Cell Disrupter (Heat-Systems-Ultrasonics, Inc., Plainview, NY), sonicating at setting 3 for 10 seconds 5 times with a one minute ice rest between sets. After clarification, immunoprecipitation was performed with 3 × 10^9^ cells of chromatin, the monoclonal anti-c-MYC antibody 9E10 (Roche Applied Science, Indianapolis, IN) and protein A sepharose CL-4B beads (GE Healthcare, Pittsburg, PA) rolling overnight at 4°C. Immune complexes were washed twice with 1 ml of lysis buffer, 1 ml of lysis buffer with 250 mM NaCl, 1 ml of ChIP wash buffer (10 mM Tris pH 8.0, 250 mM LiCl, 0.75% NP-40, 0.75% Na deoxycholate, 1 mM EDTA), and 1 ml of Tris-EDTA. DNA-protein cross-linking was reversed in 100 μl 1% SDS/Tris-EDTA at 65°C overnight. DNA was cleaned up with 50μg RNase A at 37°C for one hour then 300μg Proteinase K at 50°C for one hour. DNA was purified on Purelink PCR Purification columns (Invitrogen, Grand Island, NY) according to the manufacturer's instructions. PCR reactions (5 min 95°C, 26 times [1 min 94°C, 1 min 55°C, 1 min 72°C], 10 min 72°C, hold 4°C) were performed using HotStarTaq Plus DNA Polymerase (QIAGEN, Hilden, Germany) on 1 μl of 1/1000 eluted input (chromatin) and 1 μl of eluted immunoprecipitation. Sequences of the primers used to detect binding are available upon request. PCR fragments were separated on a 2% agarose gel and visualized by ethidium bromide.

### Accession numbers

All the demultiplexed FASTQ RNA sequence files are available from the National Center for Biotechnology Sequence Read Archive from accession SRP068917.

## Supporting Information

S1 Fig Flow cytometry data showing the importance of Swi6 and Swi4 and Msa proteins in cell cycle arrest and cell growth control during the transition to quiescence. Samples were collected over the time series of growth from log phase [8 hours) through seven days and from high density (Q) cells purified from those seven day cultures, as indicated left. Scatter plots of forward versus side light scattering and histograms of DNA fluorescence intensity are provided. Log phase DNA (8hr) shows the 1N and 2N DNA peaks that predominate in cycling cells. The peak of reduced fluorescence that accumulates late in the time course is characteristic of purified quiescent cells [15]. All strains are isogenic to BY6500 (WT, top left) and are listed in Table 1, except WT *SSD1* (BY6641) which serves as the wild type control for *swi4 SSD1* (BY7122.)
(TIF)

S2 Fig Msa1 and Msa2 are key regulators of mRNA levels in post-diauxic cells. RNA deep sequencing data for *msa1*, *msa2* and *msa1msa2* mutants are plotted against wild type as indicated from cells in the log phase of growth (left panels) and after the diauxic shift (right.) mRNA levels that differ by more than two-fold are represented by dots outside of the red lines.
(TIF)

S3 Fig Swi4 promotes cell cycle specific activation of the *HTA1* promoter driving *lacZ* transcription. (Upper panel) Histone *HTA1* promoter activity was followed for two cell cycles after release from alpha factor arrest. *CLB2* transcript levels (Lower panel) serve as a control for the loss of synchrony in each mutant strain (as indicated.) Cells were arrested in G1 with alpha factor, released and mRNAs were sampled across two cell cycles and quantified by S1 nuclease protection as previously described [34]. The invariant *ACT1* mRNA serves as a loading control. The wild type (WT) strain is isogenic with W303 *MATa*, but it contains a *LEU2* plasmid carrying *CLN2* mRNA driven by the *MET3* promoter integrated at *leu2* and a *URA3* plasmid carrying the *HTA1* promoter driving *lacZ* integrated at *ura3* (BY4438). Other strains are isogenic with this WT, except for the additional deletions of *mbp1*::*KanMx* (BY4444), *swi4*::*HIS3-965* (BY4450), or both *mbp1*::*KanMx swi4*::*HIS3-965* (BY4450.) The *MET3*-driven expression of *CLN2* is required for the viability of the *mbp1*::*KanMx swi4*::*HIS3-965* strain.
(TIF)

S4 Fig Bar graph of mRNAs more affected by *msa1msa2* than the single mutants. Transcripts listed in Table 2 are plotted on a log base 2 scale to graphically display their levels in *msa1msa2*/WT and the *msa1*/WT and *msa2*/WT.
(TIF)

S1 Table mRNA levels of known SBF and/or MBF targets in *msa* mutants. RNA sequence data for known SBF and MBF target promoters [37] is reported as a ratio of log_2_ fold difference between mutant and wild type cells as indicated. The first three measurements for each transcript are taken from log phase cells, and the second set of three are from cells that have just undergone the diauxic shift.
(DOCX)
