# Supplementary figures and images for: Msa1 and Msa2 Modulate G1-Specific Transcription to Promote G1 Arrest and the Transition to Quiescence in Budding Yeast
Source: PLoS Genet. 2016 Jun 6;12(6):e1006088. doi: 10.1371/journal.pgen.1006088 (PMC4894574; doi:10.1371/journal.pgen.1006088)

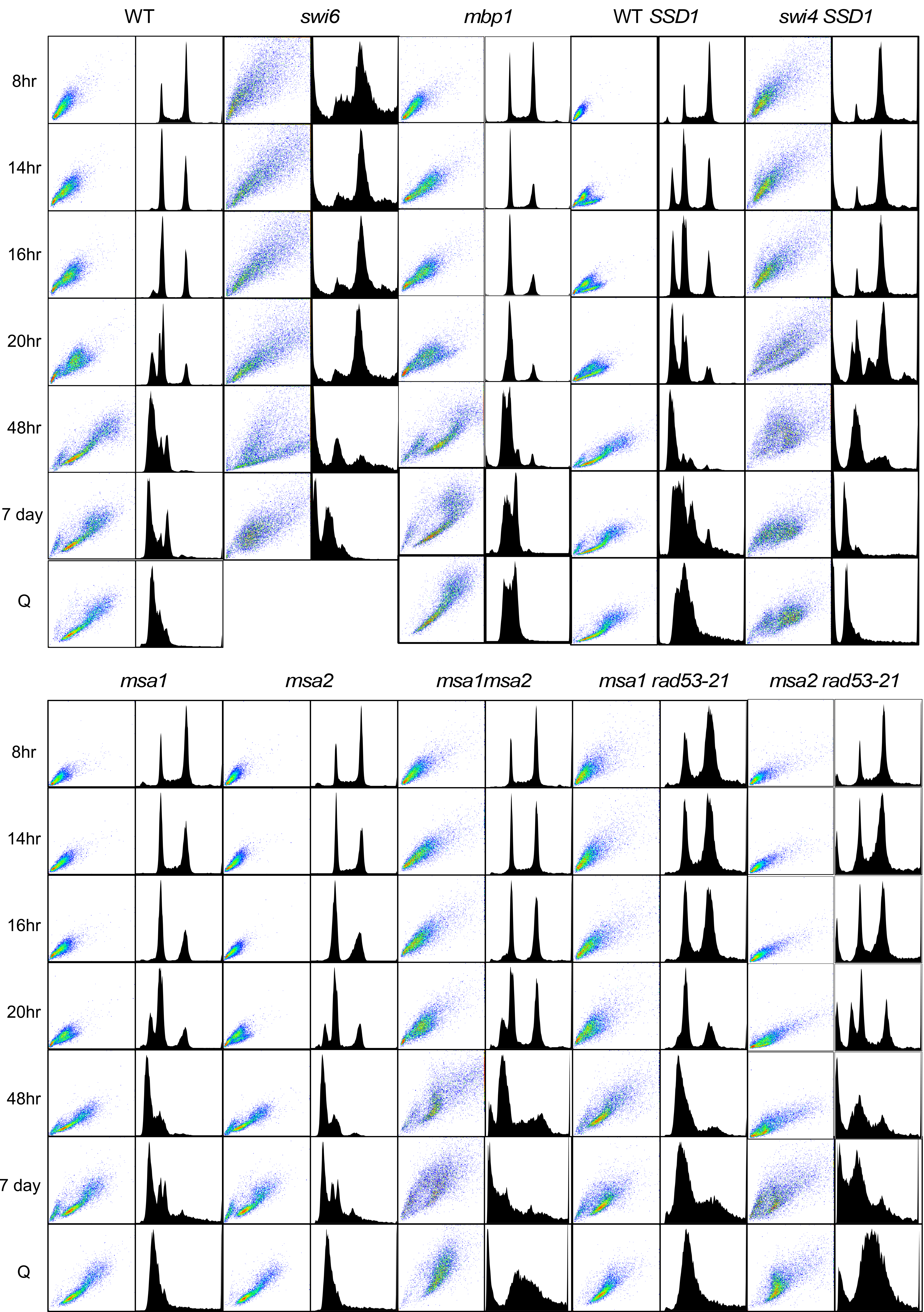

Supplement: S1 Fig — Samples were collected over the time series of growth from log phase [8 hours) through seven days and from high density (Q) cells purified from those seven day cultures, as indicated left. Scatter plots of forward versus side light scattering and histograms of DNA fluorescence intensity are provided. Log phase DNA (8hr) shows the 1N and 2N DNA peaks that predominate in cycling cells. The peak of reduced fluorescence that accumulates late in the time course is characteristic of purified quiescent cells [15]. All strains are isogenic to BY6500 (WT, top left) and are listed in Table 1, except WT SSD1 (BY6641) which serves as the wild type control for swi4 SSD1 (BY7122.) (TIF) [file pgen.1006088.s001.tif]

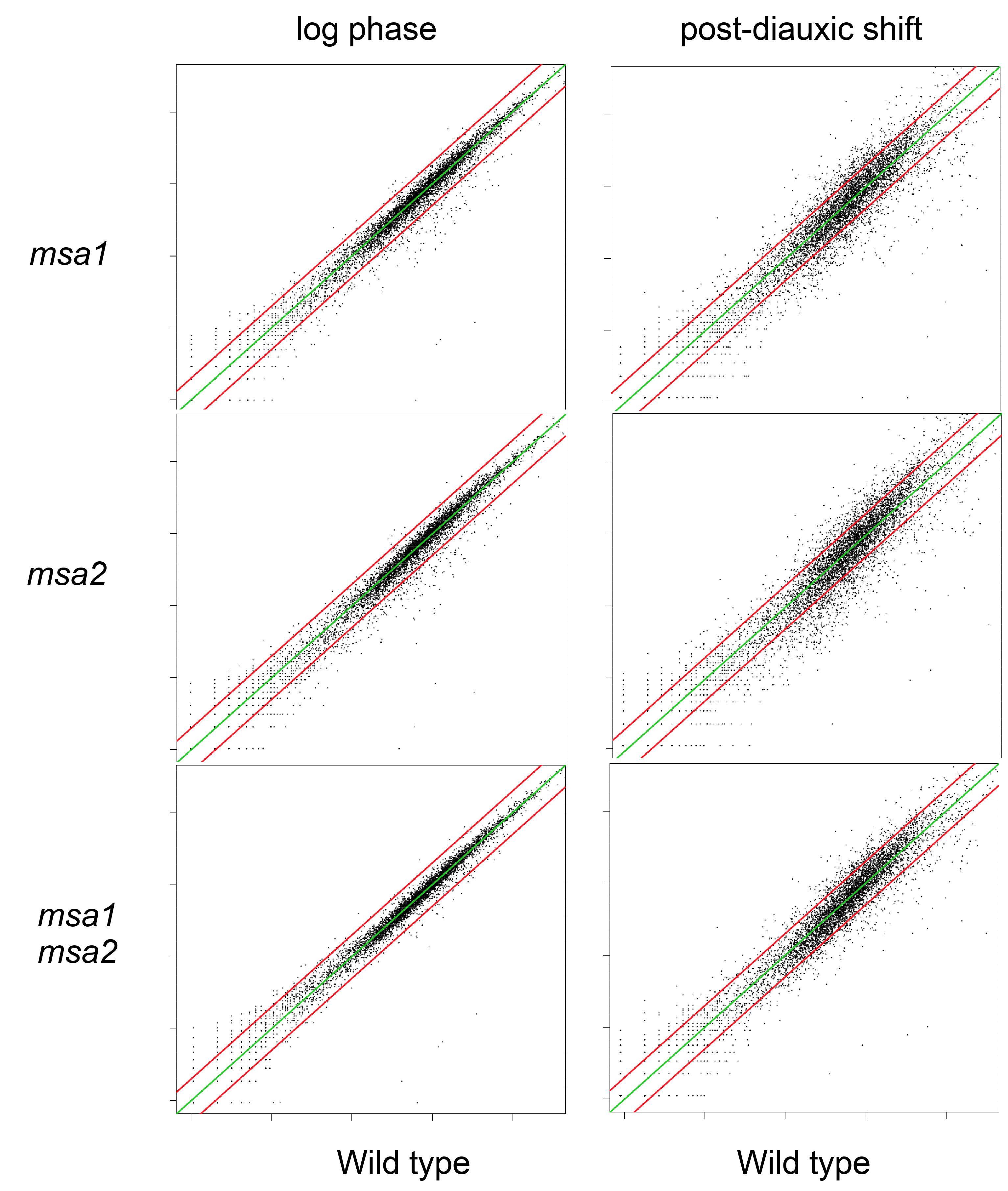

Supplement: S2 Fig — RNA deep sequencing data for msa1, msa2 and msa1msa2 mutants are plotted against wild type as indicated from cells in the log phase of growth (left panels) and after the diauxic shift (right.) mRNA levels that differ by more than two-fold are represented by dots outside of the red lines. (TIF) [file pgen.1006088.s002.tif]

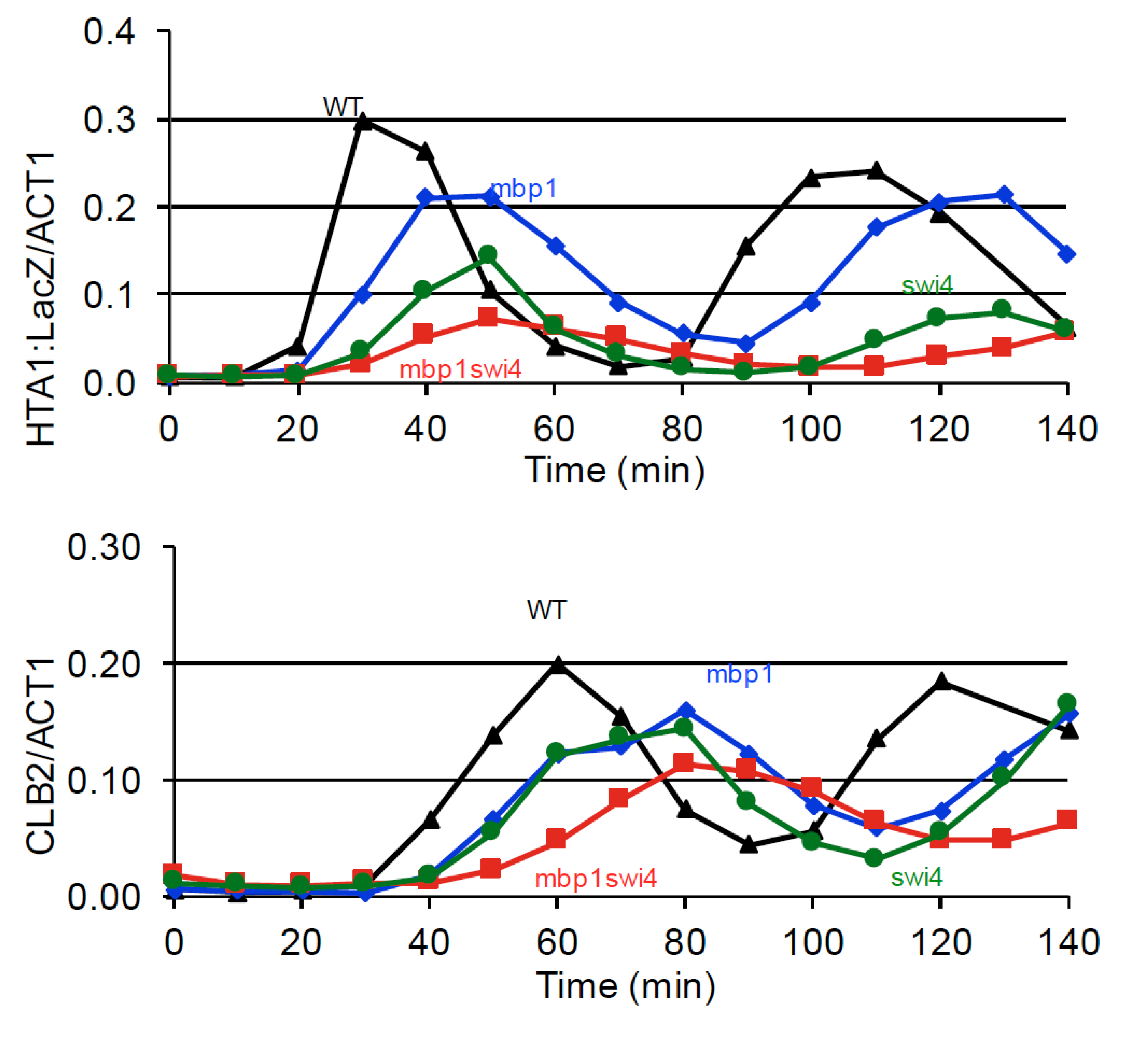

Supplement: S3 Fig — (Upper panel) Histone HTA1 promoter activity was followed for two cell cycles after release from alpha factor arrest. CLB2 transcript levels (Lower panel) serve as a control for the loss of synchrony in each mutant strain (as indicated.) Cells were arrested in G1 with alpha factor, released and mRNAs were sampled across two cell cycles and quantified by S1 nuclease protection as previously described [34]. The invariant ACT1 mRNA serves as a loading control. The wild type (WT) strain is isogenic with W303 MATa, but it contains a LEU2 plasmid carrying CLN2 mRNA driven by the MET3 promoter integrated at leu2 and a URA3 plasmid carrying the HTA1 promoter driving lacZ integrated at ura3 (BY4438). Other strains are isogenic with this WT, except for the additional deletions of mbp1::KanMx (BY4444), swi4::HIS3-965 (BY4450), or both mbp1::KanMx swi4::HIS3-965 (BY4450.) The MET3-driven expression of CLN2 is required for the viability of the mbp1::KanMx swi4::HIS3-965 strain. (TIF) [file pgen.1006088.s003.tif]

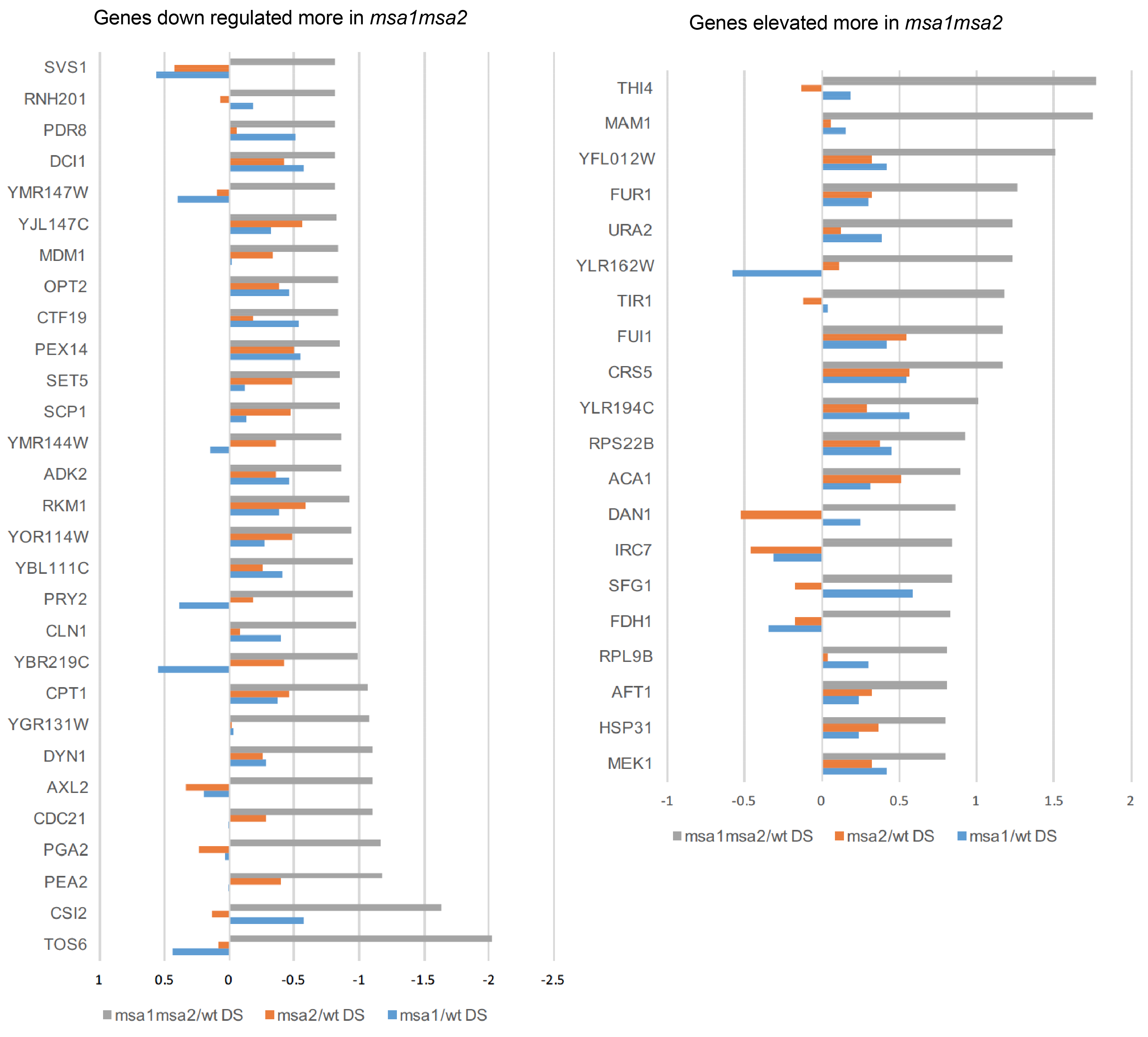

Supplement: S4 Fig — Transcripts listed in Table 2 are plotted on a log base 2 scale to graphically display their levels in msa1msa2/WT and the msa1/WT and msa2/WT. (TIF) [file pgen.1006088.s004.tif]
