# Supplementary material for: Msa1 and Msa2 Modulate G1-Specific Transcription to Promote G1 Arrest and the Transition to Quiescence in Budding Yeast
Source: PLoS Genet. 2016 Jun 6;12(6):e1006088. doi: 10.1371/journal.pgen.1006088 (PMC4894574; doi:10.1371/journal.pgen.1006088)
Supplement: S1 Table — RNA sequence data for known SBF and MBF target promoters [37] is reported as a ratio of log2 fold difference between mutant and wild type cells as indicated. The first three measurements for each transcript are taken from log phase cells, and the second set of three are from cells that have just undergone the diauxic shift. (DOCX) [file pgen.1006088.s005.docx]

Supplemental Table S1. mRNA levels for known SBF and/or MBF targets in *msa* mutants

| gene | MBF/SBF | *msa1*/wt log | *msa2*/wt log | *msa1msa2*/wt log | *msa1*/wt DS | *msa2*/wt DS | *msa1msa2*/wt DS |
| --- | --- | --- | --- | --- | --- | --- | --- |
| OGG1 | MBF | 0.20 | 0.28 | 0.18 | -2.18 | -2.48 | -1.17 |
| SLK19 | MBF | 0.02 | 0.23 | -0.15 | -1.58 | -1.50 | -1.16 |
| PDS5 | MBF | -0.35 | 0.17 | -0.55 | -1.58 | -0.98 | -1.94 |
| SEN34 | MBF | -0.10 | 0.30 | -0.15 | -1.57 | -1.93 | -1.62 |
| POL12 | MBF | 0.03 | 0.36 | 0.04 | -1.52 | -1.59 | -1.19 |
| RLF2 | MBF | 0.19 | 0.36 | 0.13 | -1.47 | -1.37 | -0.80 |
| MSH6 | MBF | -0.30 | 0.20 | -0.21 | -1.44 | -1.45 | -1.18 |
| SMC3 | MBF | -0.58 | 0.18 | -0.47 | -1.40 | -1.21 | -1.20 |
| BNR1 | MBF | 0.15 | 0.34 | -0.09 | -1.37 | -0.86 | -0.32 |
| SPH1 | MBF | 0.11 | 0.13 | -0.11 | -1.34 | -1.43 | -1.82 |
| POL32 | MBF | -0.09 | 0.07 | -0.32 | -1.24 | -1.86 | -1.05 |
| SMC5 | MBF | 0.08 | 0.34 | -0.02 | -1.23 | -0.92 | -0.72 |
| PMS1 | MBF | -0.09 | 0.33 | -0.20 | -1.10 | -0.70 | -1.16 |
| SMC1 | MBF | -0.28 | 0.21 | -0.29 | -1.05 | -0.79 | -0.97 |
| STB1 | MBF | -0.19 | 0.05 | -0.01 | -1.04 | -0.60 | -0.90 |
| BNI4 | MBF | -0.35 | -0.06 | -0.25 | -0.98 | -0.93 | -0.47 |
| TUB4 | MBF | -0.04 | 0.52 | 0.11 | -0.91 | -0.82 | -1.06 |
| RAD53 | MBF | 0.13 | 0.55 | -0.18 | -0.90 | -1.62 | -0.63 |
| ZDS2 | MBF | 0.12 | 0.29 | 0.22 | -0.85 | -1.08 | -0.72 |
| YJR030C | MBF | 0.13 | 0.38 | 0.01 | -0.85 | -0.65 | -0.96 |
| RAD5 | MBF | -0.09 | 0.18 | 0.10 | -0.84 | -0.74 | -0.70 |
| CDC45 | MBF | 0.00 | 0.21 | -0.08 | -0.80 | -0.64 | -1.36 |
| TOP2 | MBF | -0.27 | 0.14 | -0.14 | -0.79 | -0.56 | -0.79 |
| SLD2 | MBF | 0.33 | 0.58 | 0.00 | -0.77 | -0.99 | -0.74 |
| ASF1 | MBF | 0.01 | 0.28 | -0.09 | -0.74 | -0.47 | -0.39 |
| CSM3 | MBF | -0.23 | 0.40 | -0.50 | -0.71 | 0.04 | -1.06 |
| ESC8 | MBF | -0.12 | 0.11 | -0.30 | -0.70 | -0.75 | -0.95 |
| DPB2 | MBF | 0.32 | 0.58 | 0.26 | -0.69 | -0.30 | -0.57 |
| IRR1 | MBF | -0.16 | 0.21 | -0.36 | -0.68 | -0.56 | -1.12 |
| NSE4 | MBF | -0.21 | 0.30 | -0.12 | -0.68 | -0.67 | -0.50 |
| MIF2 | MBF | 0.36 | 0.59 | 0.15 | -0.67 | -0.63 | -0.04 |
| HUA2 | MBF | -0.41 | 0.20 | -0.40 | -0.65 | 0.03 | -1.28 |
| OTU2 | MBF | 0.59 | 0.61 | 0.26 | -0.60 | -0.90 | -0.30 |
| POL3 | MBF | -0.08 | 0.19 | -0.16 | -0.59 | -0.54 | -0.20 |
| MRC1 | MBF | -0.01 | 0.31 | -0.16 | -0.58 | -0.64 | -0.42 |
| CTF4 | MBF | -0.41 | 0.12 | -0.40 | -0.58 | -0.55 | -0.47 |
| RNH202 | MBF | -0.03 | 0.14 | -0.13 | -0.56 | -0.64 | -0.30 |
| YDL156W | MBF | -0.59 | 0.05 | -0.60 | -0.54 | -0.60 | -0.72 |
| SPC42 | MBF | -0.29 | -0.03 | -0.38 | -0.48 | -0.55 | -0.33 |
| PRI2 | MBF | -0.07 | 0.22 | -0.04 | -0.47 | -0.07 | -0.45 |
| ELG1 | MBF | 0.42 | 0.58 | 0.18 | -0.44 | -0.42 | -0.50 |
| RKM1 | MBF | -0.14 | 0.27 | -0.29 | -0.38 | -0.59 | -0.93 |
| MKC7 | MBF | 0.39 | 0.55 | 0.36 | -0.20 | -0.68 | -0.79 |
| SHO1 | MBF | 0.04 | -0.15 | 0.00 | -0.19 | -0.62 | -0.30 |
| RTT106 | MBF | 0.17 | 0.28 | 0.14 | -0.18 | -0.21 | -0.34 |
| GRX7 | MBF | -0.21 | -0.58 | -0.05 | -0.17 | -0.09 | -0.31 |
| RFA2 | MBF | 0.20 | 0.40 | -0.06 | -0.09 | -0.17 | -0.32 |
| SPO16 | MBF | -0.25 | 0.91 | -0.45 | -0.07 | 1.60 | 0.11 |
| CLB5 | MBF | 0.49 | 0.53 | 0.10 | -0.05 | 0.27 | 0.10 |
| TOF1 | MBF | -0.10 | 0.22 | -0.07 | -0.04 | -0.57 | -0.30 |
| YPR174C | MBF | 0.16 | 0.50 | 0.35 | -0.01 | -0.35 | -0.03 |
| RIF1 | MBF | -0.23 | 0.17 | -0.02 | -0.01 | 0.02 | -0.30 |
| CDC21 | MBF | 0.00 | 0.37 | -0.43 | 0.00 | -0.29 | -1.10 |
| MSH2 | MBF | 0.07 | 0.24 | -0.07 | 0.00 | -0.14 | -0.58 |
| CDC9 | MBF | -0.12 | 0.24 | -0.14 | 0.04 | -0.27 | -0.34 |
| MSA2 | MBF | -0.06 | -8.53 | -7.80 | 0.09 | -7.31 | -7.30 |
| SPC110 | MBF | -0.15 | 0.06 | -0.17 | 0.15 | 0.26 | -0.18 |
| RFA1 | MBF | -0.21 | 0.06 | -0.24 | 0.17 | 0.54 | 0.11 |
| DUN1 | MBF | -0.19 | -0.23 | -0.09 | 0.19 | 0.21 | -0.47 |
| DBF4 | MBF | -0.01 | 0.16 | 0.08 | 0.23 | 0.58 | 0.14 |
| YIF1 | MBF | 0.17 | 0.34 | 0.07 | 0.29 | -0.11 | -0.24 |
| YDL157C | MBF | 0.57 | 0.41 | 0.27 | 0.30 | -0.10 | 0.10 |
| ERP5 | MBF | 0.03 | 0.28 | -0.06 | 0.36 | 0.37 | 0.09 |
| ASF2 | MBF | -0.60 | -0.25 | -0.27 | 0.49 | 0.99 | 0.27 |
| ERV25 | MBF | 0.10 | 0.27 | 0.00 | 0.53 | 0.32 | 0.28 |
| RAD17 | MBF | 0.11 | -0.08 | -0.11 | 0.54 | 0.71 | 0.50 |
| RAD51 | MBF | 0.25 | 0.09 | 0.38 | 0.56 | 0.54 | 0.64 |
| BIM1 | MBF | -0.11 | -0.02 | -0.23 | 0.66 | 0.11 | 0.08 |
| BUD2 | MBF | -0.53 | -0.27 | -0.52 | 0.70 | 0.76 | 0.39 |
| YJL181W | MBF | -0.22 | -0.20 | -0.12 | 0.79 | 0.04 | 0.54 |
| ERP3 | MBF | -0.12 | 0.70 | -0.19 | 1.02 | 1.28 | 0.76 |
| YLR049C | MBF | -0.20 | -0.04 | -0.35 | 1.04 | 1.42 | 0.35 |
| YML133C | MBF | -Inf | -Inf | -Inf | -Inf | 0.32 | -Inf |
| YRF1-6 | MBF | NaN | Inf | Inf | -Inf | 0.32 | -Inf |
| POL1 | MBF/SBF | -0.16 | 0.14 | -0.27 | -1.54 | -1.33 | -1.81 |
| CBF2 | MBF/SBF | -0.11 | -0.04 | -0.07 | -1.45 | -1.87 | -1.12 |
| PDR16 | MBF/SBF | 0.04 | 0.10 | -0.13 | -1.44 | -0.97 | -0.69 |
| GIC2 | MBF/SBF | 0.28 | 0.42 | 0.10 | -1.31 | -2.00 | -1.37 |
| RTT107 | MBF/SBF | -0.15 | 0.24 | -0.35 | -1.28 | -1.02 | -0.83 |
| YOX1 | MBF/SBF | 1.05 | 0.90 | 0.23 | -1.24 | -0.69 | -0.77 |
| SKG6 | MBF/SBF | 0.25 | 0.50 | 0.37 | -1.04 | -1.57 | -1.40 |
| GIN4 | MBF/SBF | 0.06 | 0.22 | -0.06 | -1.03 | -0.71 | -1.56 |
| SMC6 | MBF/SBF | -0.08 | 0.24 | -0.02 | -0.99 | -0.73 | -0.79 |
| SPA2 | MBF/SBF | -0.12 | 0.14 | -0.14 | -0.97 | -1.05 | -0.91 |
| MNN1 | MBF/SBF | -0.80 | -0.80 | -0.74 | -0.86 | -1.15 | -1.05 |
| MCD1 | MBF/SBF | 0.33 | -0.06 | -0.55 | -0.81 | -0.60 | -0.82 |
| RSR1 | MBF/SBF | 0.29 | 0.41 | 0.39 | -0.75 | -0.71 | -0.63 |
| RAD27 | MBF/SBF | 0.13 | 0.33 | -0.04 | -0.69 | -0.66 | -1.09 |
| ALG14 | MBF/SBF | 0.17 | 0.26 | 0.24 | -0.61 | -0.23 | -0.58 |
| EXG1 | MBF/SBF | -0.49 | -0.58 | -0.29 | -0.55 | -0.89 | -0.68 |
| RNR1 | MBF/SBF | 0.16 | -0.08 | -0.26 | -0.51 | -1.32 | -1.56 |
| HCM1 | MBF/SBF | 0.04 | -0.11 | -0.34 | -0.46 | -0.63 | -0.18 |
| CLN1 | MBF/SBF | -0.74 | -0.66 | -0.74 | -0.40 | -0.08 | -0.98 |
| POL30 | MBF/SBF | 0.20 | 0.23 | 0.19 | -0.39 | -0.68 | 0.11 |
| CLB6 | MBF/SBF | 1.10 | 0.67 | -0.11 | -0.28 | 0.22 | -0.41 |
| YOR114W | MBF/SBF | -0.42 | -0.72 | -0.28 | -0.27 | -0.49 | -0.94 |
| ACM1 | MBF/SBF | 0.18 | -0.08 | -0.57 | -0.22 | 0.49 | -0.76 |
| SPT21 | MBF/SBF | 0.40 | 0.61 | 0.29 | -0.21 | -0.54 | -0.04 |
| YBR071W | MBF/SBF | -0.05 | 0.03 | 0.09 | -0.17 | 0.10 | -0.47 |
| RFA3 | MBF/SBF | 0.33 | 0.30 | 0.11 | -0.04 | -0.02 | -0.39 |
| HSL1 | MBF/SBF | -0.03 | 0.13 | -0.03 | -0.01 | -0.38 | -0.66 |
| YMR144W | MBF/SBF | -0.09 | 0.06 | -0.03 | 0.14 | -0.35 | -0.86 |
| AXL2 | MBF/SBF | -0.01 | 0.10 | 0.20 | 0.20 | 0.33 | -1.10 |
| SWE1 | MBF/SBF | 0.17 | 0.22 | -0.04 | 0.32 | 0.56 | -0.56 |
| PDS1 | MBF/SBF | -0.16 | -0.28 | -0.51 | 0.34 | -0.08 | -0.74 |
| OCH1 | MBF/SBF | -0.93 | -0.66 | -0.40 | 0.40 | 0.66 | 0.47 |
| SUR2 | MBF/SBF | -0.79 | -0.69 | -0.13 | 0.45 | 0.13 | 0.66 |
| CRH1 | MBF/SBF | 0.20 | 0.26 | 0.47 | 0.74 | 0.49 | 0.02 |
| SCW10 | MBF/SBF | -0.22 | -0.18 | 0.17 | 0.80 | 0.45 | -0.21 |
| SWI4 | MBF/SBF | 0.07 | 0.45 | 0.42 | 1.32 | 1.17 | 1.28 |
| HHF2 | MBF/SBF | 0.38 | 0.32 | 0.22 | 2.78 | 2.74 | 2.25 |
| CAF120 | SCB | -0.15 | 0.08 | -0.05 | -0.96 | -0.56 | -0.27 |
| ALK2 | SCB | 0.21 | 0.31 | 0.14 | -0.66 | -1.24 | -1.47 |
| CSI2 | SCB | 0.29 | 0.43 | 0.10 | -0.57 | 0.13 | -1.63 |
| MSB2 | SCB | -0.04 | 0.05 | 0.08 | -0.57 | -0.63 | -0.55 |
| YOR246c | SCB | 0.10 | 0.04 | 0.07 | -0.46 | -0.68 | -0.47 |
| YJR054w | SCB | 0.20 | 0.25 | -0.02 | -0.37 | -0.86 | -0.43 |
| CSE4 | SCB | 0.18 | 0.19 | -0.04 | -0.30 | -0.50 | -0.56 |
| SVL3 | SCB | -0.19 | -0.04 | -0.11 | -0.29 | -0.15 | -0.17 |
| PCL2 | SCB | 0.27 | 0.60 | 0.19 | -0.22 | 0.20 | -0.54 |
| HO | SCB | -0.37 | -0.60 | -2.73 | -0.04 | 0.03 | 0.04 |
| YMR304C-A | SCB | -0.11 | 0.32 | 0.07 | -0.02 | -0.35 | -0.88 |
| SPC105 | SCB | -0.09 | 0.05 | -0.23 | 0.02 | -0.27 | -0.22 |
| YHR173c | SCB | 0.35 | 0.62 | 0.24 | 0.03 | -1.07 | -1.25 |
| BBP1 | SCB | 0.01 | 0.05 | -0.01 | 0.05 | -0.10 | 0.33 |
| VRG4 | SCB | -0.01 | -0.01 | -0.11 | 0.06 | -0.75 | -0.59 |
| SUR1 | SCB | -0.91 | -0.83 | -0.57 | 0.13 | 0.45 | 0.29 |
| MGM101 | SCB | 0.19 | 0.03 | 0.07 | 0.13 | 0.16 | 0.00 |
| YPS3 | SCB | -0.74 | -0.68 | 0.25 | 0.15 | 0.28 | 0.45 |
| SMF2 | SCB | -0.47 | -0.46 | -0.11 | 0.19 | 0.34 | 0.16 |
| NRM1 | SCB | 0.65 | 0.57 | 0.23 | 0.24 | 0.37 | -0.24 |
| NUD1 | SCB | -0.09 | 0.05 | -0.20 | 0.28 | 0.59 | 0.10 |
| SCP160 | SCB | -0.31 | -0.22 | -0.11 | 0.28 | -0.55 | -0.22 |
| DUT1 | SCB | 0.15 | 0.19 | -0.05 | 0.29 | -0.13 | -0.31 |
| KTR1 | SCB | -0.37 | -0.30 | -0.16 | 0.29 | 0.27 | 0.11 |
| TOS4 | SCB | 0.12 | 0.04 | -0.37 | 0.32 | 0.24 | -0.05 |
| YSY6 | SCB | 0.12 | 0.27 | 0.19 | 0.36 | -0.54 | -0.04 |
| STU2 | SCB | -0.05 | 0.10 | -0.08 | 0.37 | -0.24 | -0.43 |
| PMI40 | SCB | -0.04 | 0.09 | -0.44 | 0.38 | 0.19 | -0.32 |
| PRY2 | SCB | 0.42 | 0.41 | 0.27 | 0.38 | -0.18 | -0.95 |
| PXL1 | SCB | -0.16 | 0.13 | -0.03 | 0.40 | 0.02 | -0.13 |
| SKM1 | SCB | -0.97 | -1.00 | -0.03 | 0.43 | 0.27 | -0.03 |
| TOS6 | SCB | 0.31 | 0.11 | -0.43 | 0.43 | 0.09 | -2.03 |
| SPC29 | SCB | -0.37 | 0.15 | -0.06 | 0.47 | 0.45 | -0.53 |
| HTZ1 | SCB | 0.06 | 0.02 | -0.03 | 0.54 | 0.72 | 0.32 |
| SVS1 | SCB | -0.17 | 0.02 | -0.30 | 0.57 | 0.42 | -0.81 |
| YOL019w | SCB | -0.14 | 0.00 | -0.11 | 0.61 | 0.42 | -0.55 |
| HHO1 | SCB | 0.12 | 0.06 | -0.26 | 0.65 | 0.78 | 0.22 |
| GAS1 | SCB | -0.17 | -0.03 | -0.13 | 0.67 | -0.06 | -0.41 |
| PMT2 | SCB | -0.11 | 0.01 | -0.02 | 0.68 | 0.42 | 0.45 |
| DTD1 | SCB | 0.02 | 0.09 | -0.25 | 0.72 | 0.30 | -0.30 |
| HTA1 | SCB | -0.01 | 0.01 | -0.17 | 0.79 | 0.98 | 0.80 |
| WSC2 | SCB | 0.14 | 0.17 | 0.11 | 0.85 | 0.70 | -0.14 |
| TOS1 | SCB | -0.17 | -0.18 | -0.14 | 0.91 | 0.54 | 0.11 |
| SIM1 | SCB | -0.35 | -0.34 | -0.06 | 0.92 | 0.79 | 0.06 |
| CLN2 | SCB | -0.13 | 0.05 | -0.29 | 0.99 | 1.14 | -0.43 |
| NDD1 | SCB | 0.19 | 0.12 | 0.14 | 1.14 | 1.02 | 0.42 |
| ECM33 | SCB | -0.32 | -0.27 | -0.24 | 1.16 | 1.02 | 0.88 |
| ERP2 | SCB | 0.04 | 0.13 | -0.07 | 1.25 | 0.72 | 0.71 |
| HHT2 | SCB | 0.30 | 0.25 | 0.19 | 1.28 | 1.35 | 1.07 |
| ERG4 | SCB | 0.59 | 0.52 | 0.61 | 1.29 | 0.91 | 0.82 |
| PGM1 | SCB | -0.03 | 0.06 | 0.02 | 1.37 | 1.05 | 0.28 |
| HTB1 | SCB | 0.07 | 0.05 | -0.06 | 1.42 | 1.55 | 1.29 |
| HHF1 | SCB | 0.08 | -0.05 | -0.15 | 1.72 | 1.25 | 0.96 |
| PSA1 | SCB | -0.11 | -0.17 | -0.25 | 2.15 | 1.97 | 1.29 |
| CIS3 | SCB | -0.12 | 0.01 | -0.06 | 2.20 | 1.49 | 0.79 |
| SRL1 | SCB | 0.39 | 0.23 | 0.19 | 2.54 | 2.40 | 1.81 |
| HTB2 | SCB | 0.35 | 0.22 | 0.06 | 3.28 | 2.33 | 1.57 |
| HTA2 | SCB | 0.38 | 0.23 | 0.12 | 3.89 | 3.55 | 2.70 |

Expression levels of all known gene targets of MBF or SBF or both expressed as a log base 2 ratio of mutant over wild type during log phase (log) or after the diauxic shift (DS.)
